# Translationally controlled tumor protein promotes liver regeneration by activating mTORC2/AKT signaling

**DOI:** 10.1038/s41419-020-2231-8

**Published:** 2020-01-23

**Authors:** Zhibin Lin, Xuan Zhang, Jianlin Wang, Wei Liu, Qi Liu, Yuchen Ye, Bin Dai, Dongnan Guo, Pengcheng Zhang, Peijun Yang, Ruohan Zhang, Lin Wang, Kefeng Dou

**Affiliations:** Department of Hepatobiliary Surgery, Xijing Hospital, The Fourth Military Medical University, Xi’an, China

**Keywords:** Cell biology, Molecular biology

## Abstract

Translationally controlled tumor protein (TCTP), which is a protein characterized by its potent proliferation promoting activity, has been well studied in the area of growth and tumorigenesis. However, the specific role of TCTP in liver regeneration (LR) and its underlying mechanism remains unclear. In order to investigate the contribution of TCTP during LR, heterozygous TCTP mice were generated, and a mimic LR model was applied to TCTP-knockdown (KD) hepatic cell lines. The results revealed that TCTP-KD impaired LR in mice, and manifested as the following aspects: delayed proliferation of hepatocytes, accompanied by disruption of the mRNA expression of markers of the cell cycle, degenerated lipid metabolism, and abnormal immune response. Furthermore, it was found out that TCTP activated PI3K/AKT signaling by regulating mTORC2. Lastly, the increasing rate of serum TCTP positively correlated to the recovery of alanine aminotransferase (ALT) and aspartate aminotransferase (AST) after liver resection in humans. In summary, the present study is the first to reveal the crucial role of intracellular TCTP in LR.

## Introduction

Liver regeneration (LR) is the process through which the liver is able to replace lost liver tissue from the compensatory hypertrophy and hyperplasia of remaining hepatocytes. It has been recognized that 25% of the original liver mass can regenerate back to its full size after surgical resection or chemical insult^1,2^. LR is relevant in clinical scenarios. For instance, the successes of both partial liver resection and living donor liver transplant require prosperous LR. However, compromised LR in these settings result in small for size syndrome (SFSS), and causes the prolongation of intensive care stays, or even the death of patients^3^. Therefore, an increased understanding of the LR process has significant benefit in the treatment of all kinds of liver failure, and may shed light on the development of cancer within the cirrhotic liver.

Translationally controlled tumor protein (TCTP), which is also referred to as P23, fortilin, or histamine releasing factor (HRF), is a highly conserved and multifunctional protein^4^. TCTP has been proven to be highly associated with various cellular processes, including cell growth, development, apoptosis, DNA repair, immune response, malignant transformation, and tumor reversion^4–7^. One of the best-characterized functions of TCTP is its proliferation promoting activity. Researchers have found that the downregulation of *Drosophila* TCTP (*dTCTP*) reduces the proliferative cell number and organ size^8^. Remarkably, the function of TCTP in proliferation regulation remains conserved among species, since both *Arabidopsis* TCTP (*AtTCTP*) and human TCTP (*hTCTP*) can rescue *dTCTP* knockout-induced cell proliferation defects^8–10^.

Despite preliminary studies that demonstrated that the mRNA of TCTP in liver tissue increases at the early stage of rat LR^11^, and that extracellular TCTP can serve as a cytokine-like protein to facilitate LR in rats^12^, the underlying molecular mechanism by which TCTP regulates LR has not been illustrated at present. In particular, TCTP has been identified to biologically express and function within the cytoplasm^4^. Hence, the impact of intracellular TCTP on hepatocytes in LR needs to be investigated. In the present study, TCTP^+/−^ transgenic mice (homozygous is embryonically lethal^6^) and TCTP-KD hepatic cell lines were used to explore the role of intracellular TCTP, and delineate the specific regulatory mechanisms of TCTP in LR, aiming to provide treatment strategies for clinical liver regenerative disorders.

The present study is the first to demonstrate that the deletion of TCTP severely mitigates the progression of LR, hinting the crucial impact of TCTP on the development of LR. It is noteworthy that the positive impact of TCTP on LR depends on its regulation of mTORC2, which subsequently phosphorylates AKT. In addition, it was also verified that serum TCTP levels can reflect the recovery of liver function in patients following partial hepatectomy.

## Results

### TCTP was significantly induced during LR in wild-type mice

**Fig. 1:**
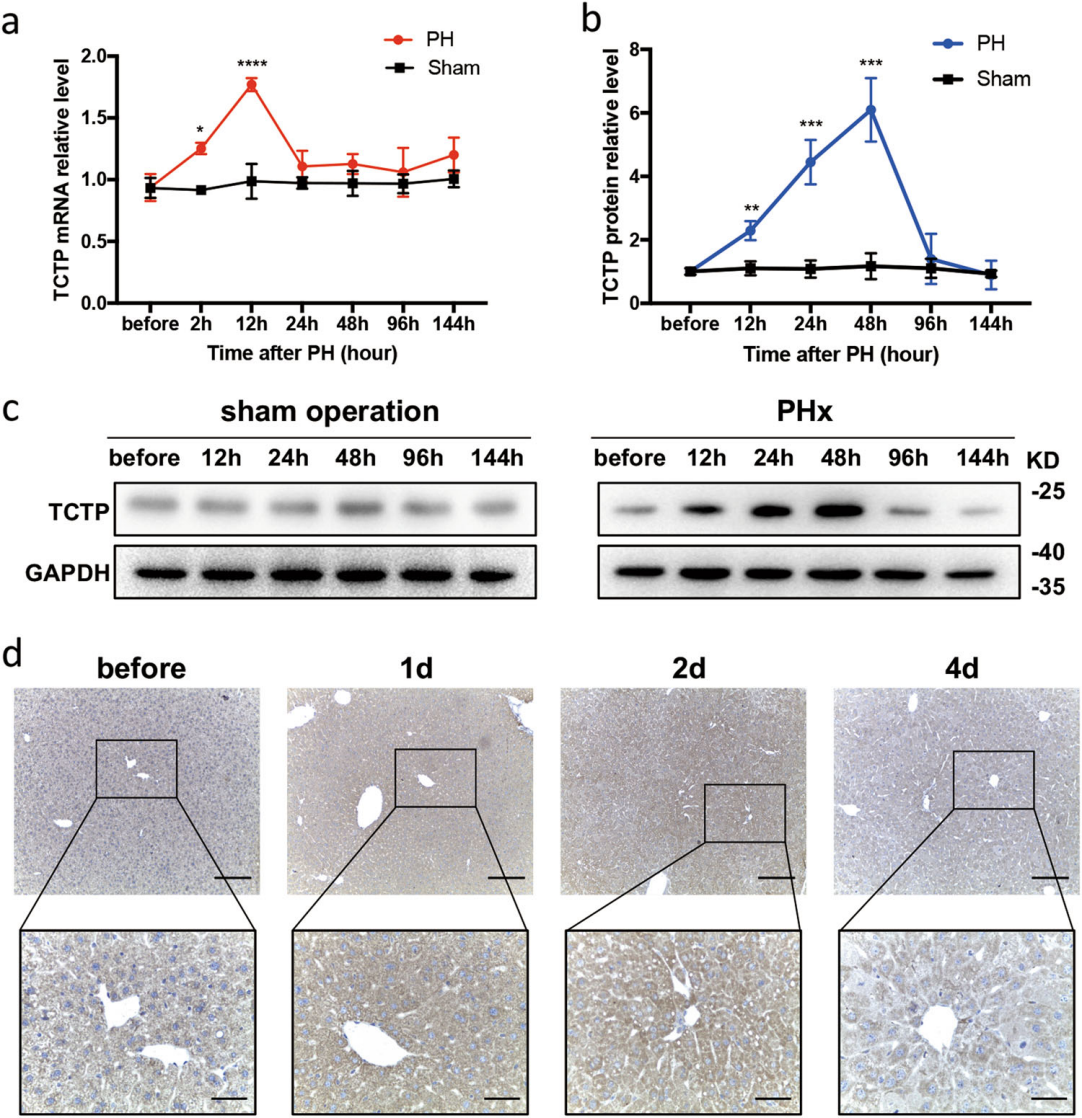
TCTP was strikingly induced during liver regeneration (LR) in wild-type mice. **a** The RT-PCR analysis of the TCTP mRNA expression in livers obtained from the PHx group (PH) and sham operation group (Sham) at the indicated time points of LR, and GAPDH was used as a reference gene. **b** Western blot analysis of the TCTP protein expression in livers obtained from the PH group and Sham group at the indicated time points of LR. The results were presented as mean ± standard deviation (SD). *n* *=* 5. *P < 0.05; **P < 0.01; ***P < 0.001; ****P < 0.0001 vs. control (Two-way ANOVA followed by Tukey’s test). **c** Representative western blot analysis of the TCTP protein expression in livers obtained from the PH group and Sham group at the indicated time points of LR. **d** Representative IHC staining of TCTP (brown) on liver sections at the indicated time points of LR; Scale bars: 200 μm (upper) and 50 μm (lower).

In order to observe the expression of TCTP in LR, 70% partial hepatectomy (PHx) was performed on wild-type C57BL/6J mice, and the TCTP level was measured at indicated time points. Remarkably, the mRNA expression of TCTP started to ascend at 2 h post-PHx, peaked at 12 h, and descended to its baseline in 48 h, when compared to the control group (Fig. 1a). Meanwhile, the TCTP protein gradually increased, reached its maximum at 48 h, and dropped to its baseline at 96 h (Fig. 1b, c). It could be observed that the time when TCTP reached its maximal expression was in accordance with the time when the peak of the DNA synthesis in hepatocytes occurred, which was at approximately 40 h after PHx, and this was earlier than that in non-parenchymal cells^13^. Accordingly, it was speculated that TCTP is mainly induced in parenchymal cells, which was evidenced by the present immunohistochemistry (IHC) results (Fig. 1d). Briefly, TCTP was overexpressed in hepatocytes at the early stage of LR.

### LR was significantly inhibited in TCTP^+/−^ mice

**Fig. 2:**
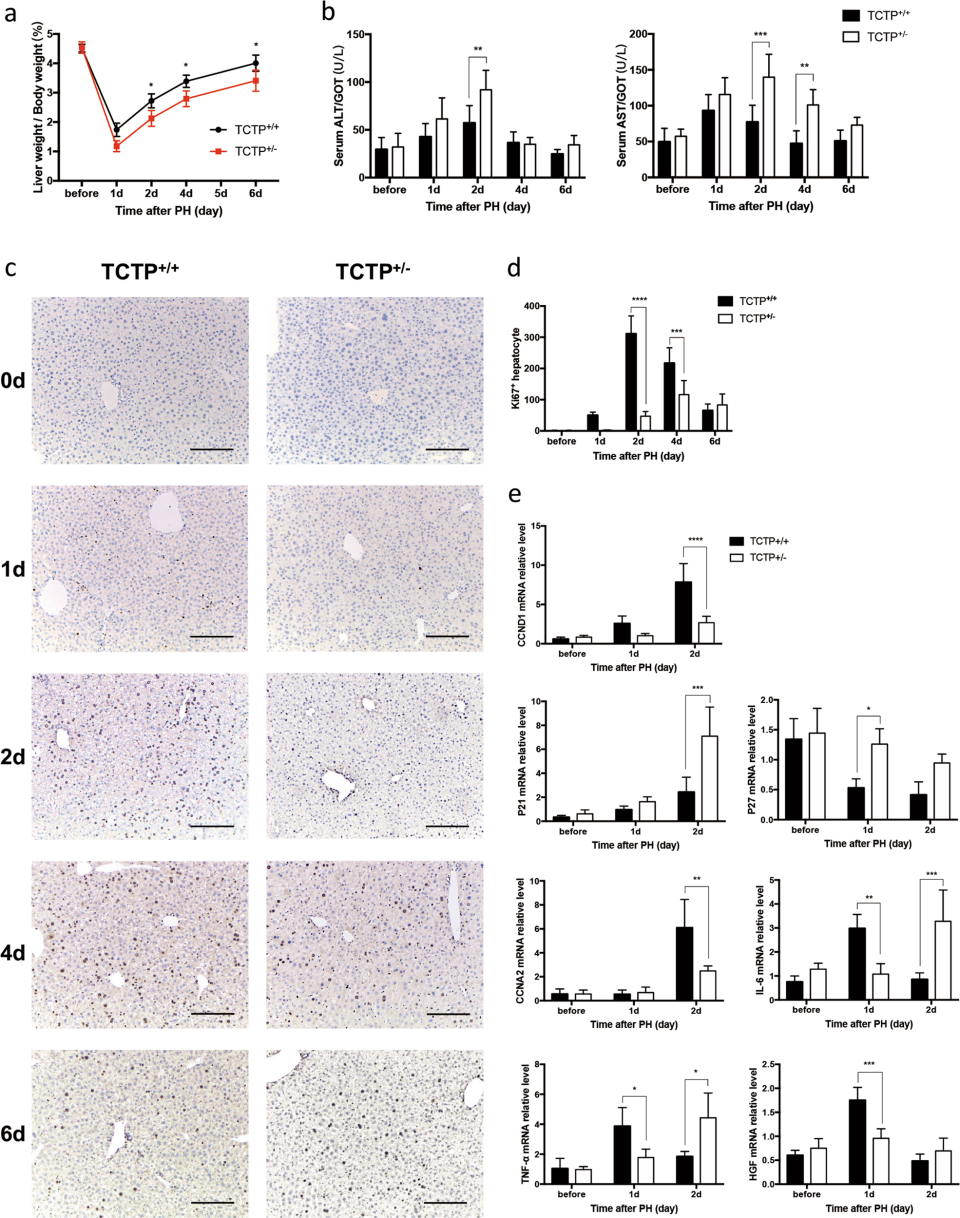
Liver regeneration (LR) was inhibited in TCTP^+/−^ mice. **a**, **b** The liver weight/body weight ratio (**a**), and serum ALT and AST levels (**b**) of TCTP^+/+^ and TCTP^+/−^ mice were measured at the indicated time points during LR. **c** Representative IHC staining of Ki67 (brown) on liver sections obtained from TCTP^+/+^ and TCTP^+/−^ mice at the indicated time points of LR; Scale bars: 200 μm. **d** The number of Ki67-positive hepatocytes was counted and used as an indicator to compare with the regenerative ability of livers obtained from TCTP^+/+^ and TCTP^+/−^ mice. **e** The RT-PCR analysis for the mRNA expression level of, CCND1, P21, P27, CCNA2, IL-6, TNF-α and HGF in livers obtained from TCTP^+/+^ and TCTP^+/−^ mice at the indicated time points of LR. The results were presented as mean ± standard deviation (SD). *n* = 5. *P < 0.05; **P < 0.01; ***P < 0.001; ****P < 0.0001 vs. control (Two-way ANOVA followed by Tukey’s test).

In order to verify the impact of TCTP on PHx-induced LR, TCTP^+/−^ mice were generated using gene targeting technology (Supplementary Fig. S1). Compared with the control group, the liver-to-body weight ratio of TCTP^+/−^ mice significantly decreased within 6 days post-PHx (Fig. 2a). It is noteworthy that relatively higher serum levels of alanine transaminase (ALT) and aspartate transaminase (AST) were observed in TCTP^+/−^ mice at each time point (Fig. 2b), indicating the delayed recovery of liver function. The Ki67 staining in the control littermates revealed that the cell proliferation in LR peaked at 48 h post-PHx. Nevertheless, the cell proliferation peak appeared at 96 h in TCTP^+/−^ mice, and exhibited a subdued proliferation efficacy, when compared to the control group (Fig. 2c, d). Thus, it could be concluded that the downregulation of TCTP mitigates cell proliferation during the LR process.

On the basis of these above findings, the alteration of the cell cycle, which can influence cell proliferation, was further investigated. It was found that CCND1, which promotes cell cycle progression in the G1 phase by sequestering p21^CIP1^ and p27^KIP1^^14^^,^, was inhibited in TCTP^+/−^ mice, resulting in the increase in p21 and p27 mRNA expression. Meanwhile, CCNA2, which is known as a key marker of the S phase^14^, exhibited a decline in mRNA level in TCTP^+/−^ mice, when compared to the control group (Fig. 2e). Therefore, it could be considered that the deletion of TCTP might prevent hepatocytes from entering the cell cycle.

In addition, it was also observed that the transcription of some pro-inflammatory cytokines and growth factors were crucial for the initiation of the cell cycle^13^. As presented in Fig. 2e, the mRNA expression of IL-6 and TNF-α was delayed in TCTP^+/−^ mice, and HGF mRNA also appeared to be lower in TCTP^+/−^ mice, when compared to the control group. Consequently, the knockdown of TCTP not only alleviated the inflammatory cytokines induced by PHx, but also restricted the production of indispensable growth factors, resulting in an impaired LR in TCTP^+/−^ mice.

### TCTP-KD disturbs lipid metabolism and immune response during LR

**Fig. 3:**
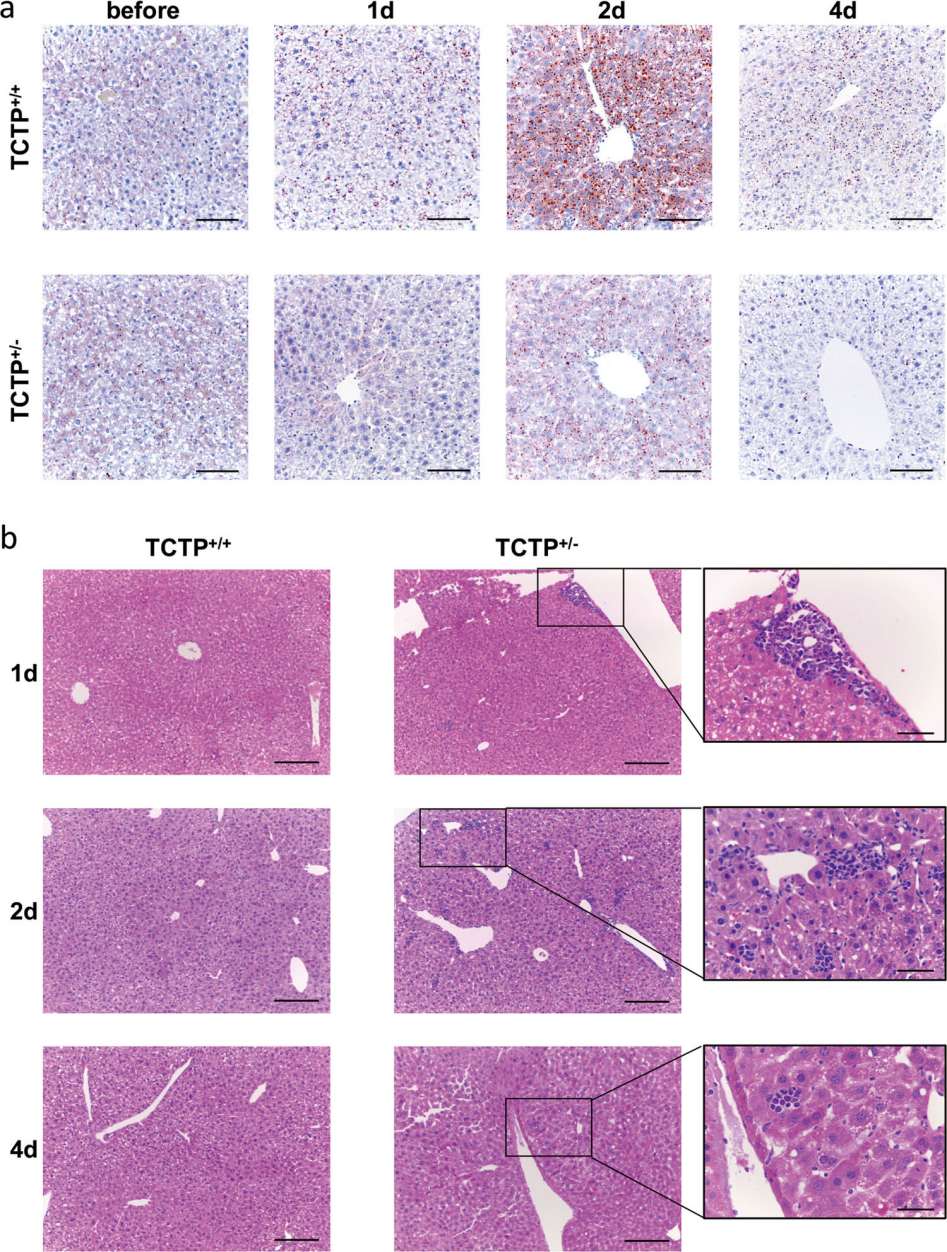
TCTP-KD disrupts lipid metabolism and immune response during liver regeneration (LR). **a**–**b** Representative histological panels of the Oil Red O staining (red, **a**) and H&E staining (**b**) in liver sections obtained from TCTP^+/+^ and TCTP^+/−^ mice at the indicated time points after PHx; Scale bars: 100 μm (**a**), 200 μm, and 50 μm (**b**); *n* = 5.

Transient steatosis occurs at the early stage of LR, and the formation of lipid droplets is indispensable for hepatocyte proliferation^15–17^. In order to observe the impact of TCTP loss on hepatic lipid metabolism, Oil Red O staining was performed to inspect the hepatic lipid accumulation in TCTP^+/−^ mice following PHx. The results revealed that lipid droplets gradually formed in wild-type hepatocytes, and peaked at post-operative day (POD) 2. In contrast, the accumulation of lipid droplets in TCTP^+/−^ mice decreased on POD2, and almost disappeared on POD4 (Fig. 3a).

In particular, the evident local infiltration of lymphocytes in TCTP^+/−^ regenerative liver tissues at different time points was observed (e.g. on POD2), which was absolutely distinct from that in the control group (Fig. 3b and Supplementary Fig. S2). Accordingly, it was assumed that the knockdown of TCTP might have caused the abnormal immune activation in the process of LR.

### TCTP activates PI3K/AKT signaling during LR

**Fig. 4:**
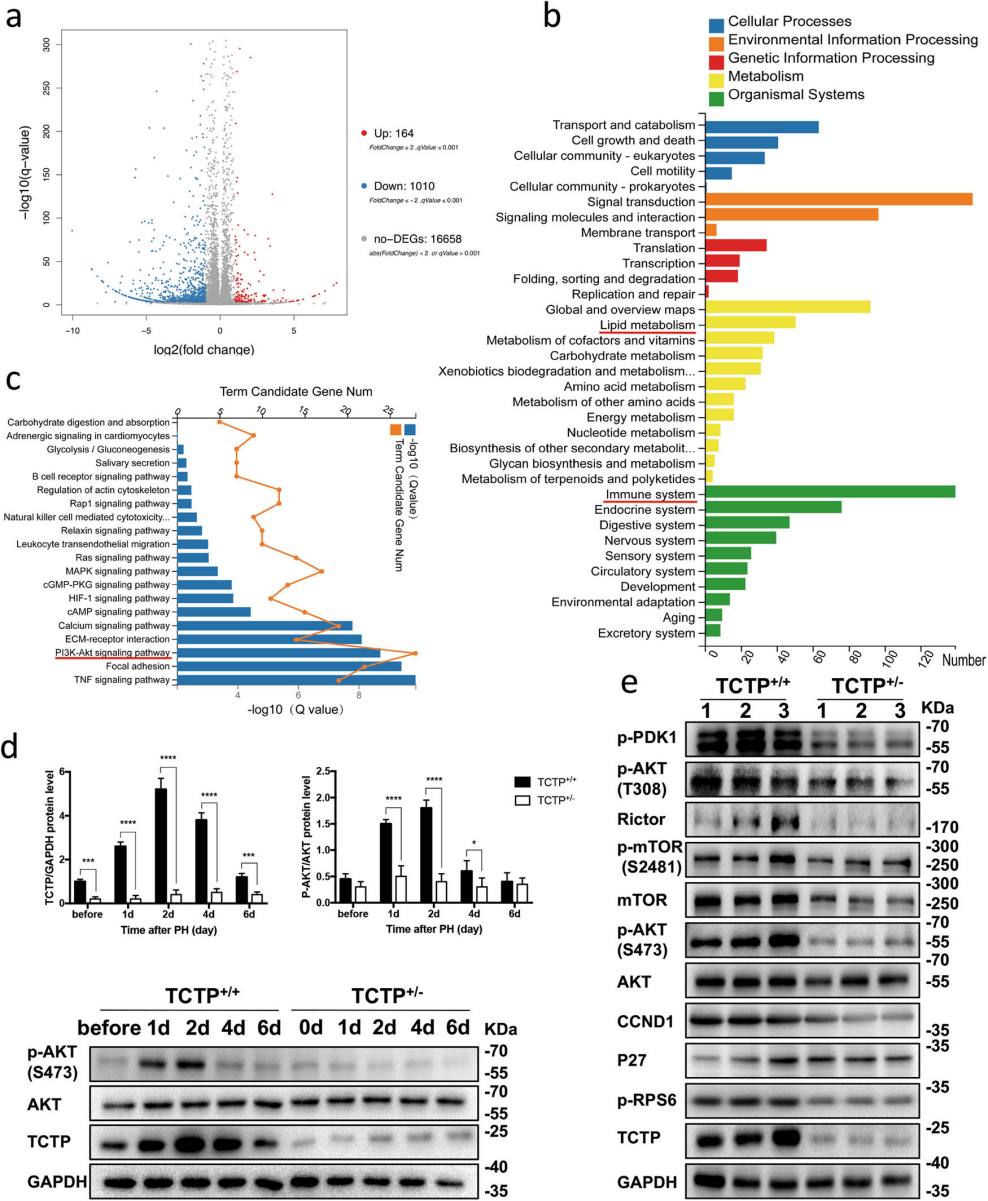
TCTP activates PI3K/AKT signaling during liver regeneration (LR). **a** A volcano plot shows the profile of differentially expressed genes (DEGs) analyzed by RNA sequencing in TCTP-KD primary hepatocytes (fold change > 2.0 and *P*-value < 0.05). **b** The KEGG pathway enrichment analysis shows the distribution of DEGs in five modules. **c** The KEGG pathway analysis of DEGs participating in the signal transduction. **d** The western blot analysis for the expression of TCTP, AKT and p-AKT^Ser473^ proteins in livers obtained from TCTP^+/+^ and TCTP^+/−^ mice at the indicated time points during LR. GAPDH was probed as a loading control (lower). The average gray value of each blot was quantified using the Image Lab^TM^ software (upper); *n* = 4. The results were presented as mean ± standard deviation (SD). *P < 0.05; ****P < 0.0001 vs. control (Two-way ANOVA followed by Tukey’s test). **e** Western blot analysis for the expression of PI3K/AKT pathway-related proteins in livers obtained from three randomly chosen TCTP^+/+^ mice and three randomly chosen TCTP^+/−^ mice at day two after PHx.

RNA sequencing was conducted to identify genes modified by the TCTP-KD during LR. The results revealed that 164 genes were upregulated and 1010 genes were downregulated after TCTP-KD (Fig. 4a). The pathway enrichment analysis of differentially expressed genes (DEGs) revealed that the expression of genes involved in lipid metabolism and the immune system was modified after TCTP-KD (Fig. 4b). This partly explains the abnormal lipid metabolism and immune response in TCTP^+/−^ mice after PHx. The signal transduction analysis revealed that PI3K/AKT signaling possessed the largest number of DEGs, hinting its dominant role in LR (Fig. 4c). In wild-type mice, phospho-AKT (p-AKT^Ser473^) gradually increased after PHx, peaked at 48 h, and declined to baseline at 96 h, which was fairly consistent with the alteration of TCTP during LR (Fig. 4d). In contrast, p-AKT^Ser473^ remained unchanged in TCTP^+/−^ mice (Fig. 4d). Accordingly, the expression of PI3K/AKT pathway-related proteins at 48 h post-PHx was compared, and it was found that some upstream proteins of AKT, such as p-PDK1 (to phosphorylate AKT^Thr308^ ^18^), mTOR (to form the core of both mTORC1 and mTORC2), RICTOR (a typical subunit of mTORC2^19^) and p-mTOR^Ser2481^ (as activated mTORC2^19^ to phosphorylate AKT^Ser473^), had a lower expression in TCTP^+/−^ mice (Fig. 4e), although the expression of PTEN (to regulate p-AKT^18^) appeared to be the same in the two groups (Supplementary Fig. S3b). Subsequently, p-AKT^Thr308^ and p-AKT^Ser473^ were observed to be reduced in TCTP^+/−^ mice (Fig. 4e). In addition, the level of some typical downstream substrates of AKT, such as p-RPS6 (a readout of mTORC1 activity^20^), P27 and cyclinD1^21^, also changed in TCTP^+/−^ mice (Fig. 4e). These above findings were also supported by the IHC assay (Supplementary Fig. S3a).

### TCTP and p-AKT proteins were significantly increased in an in vitro LR model

**Fig. 5:**
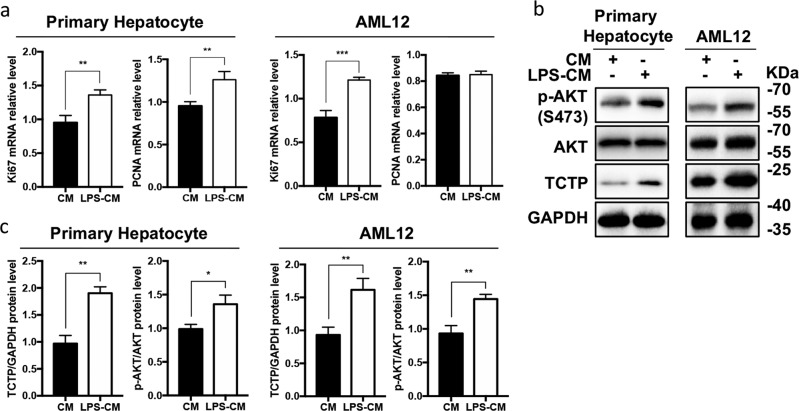
TCTP and p-AKT proteins were significantly increased in the in vitro liver regeneration (LR) model. **a** The RT-PCR analysis for the mRNA expression of PCNA and Ki67 in primary hepatocytes and the AML12 cell line treated with CM or LPS-CM, in order to testify the efficiency of the LPS-CM model. GAPDH was used as a reference gene. The LPS-CM (or CM) was obtained from LPS-treated (or PBS-treated) RAW 264.7 macrophages (20 μg/mL for 2 h), and the hepatocytes were harvested after incubation with LPS-CM (or CM) for 2 h. **b** Representative western blot analysis for the protein expression of TCTP, AKT and p-AKT^Ser473^ in the LPS-CM (or CM) treated hepatocytes. **c** The average gray value of each blot was quantified using the Image Lab^TM^ software. All experiments were conducted in triplicate. The results were presented as mean ± standard deviation (SD); **P < 0.01; ***P < 0.001 *vs*. control (two-tailed Student’s *t* test).

An in vitro LR model was applied to primary hepatocytes and the AML12 hepatic cell line to exclude the interference from non-parenchymal cells, and verify the relationship between TCTP and PI3K/AKT signaling. Briefly, hepatocytes were treated with conditioned medium obtained from either lipopolysaccharide (LPS)-treated mouse macrophages RAW 264.7 (LPS-CM), or phosphate buffer solution (PBS)-treated RAW 264.7 cells (CM), in order to simulate the initiation stage of LR^13,22^. To justify this model, the in vitro cell proliferation was observed, and the transcription of PCNA and Ki67 was checked. The results revealed that the mRNA of PCNA and Ki67 exhibited an increase in LPS-CM-treated hepatocytes (Fig. 5a). Meanwhile, the expression of TCTP and p-AKT^Ser473^ proteins were also verified (Fig. 5b, c), which was in accordance with the in vivo results. Hence, the LPS-CM model, which was proven to be able to simulate LR, was used in the subsequent experiments.

### TCTP facilitates the proliferation of hepatocytes by activating PI3K/AKT signaling

**Fig. 6:**
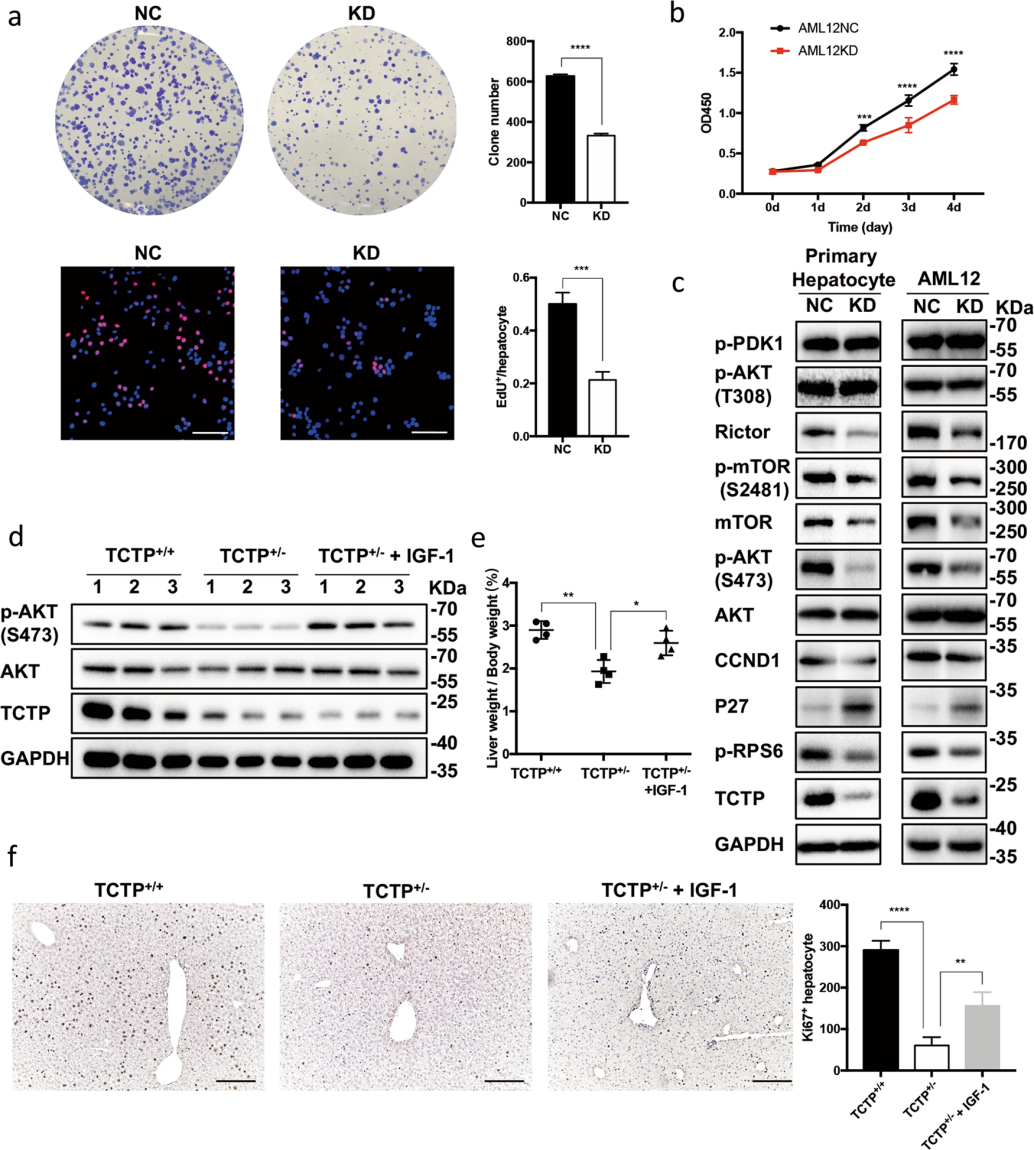
TCTP facilitates hepatocyte proliferation by activating the PI3K/AKT signaling. **a** Colony formation assays and EdU assays were performed on AML12-NC and AML12-TCTPKD cells. For colony formation assays, cells were seeded in 6-well plates (1 × 10^3^ cells/well) and incubated with LPS-CM for 10 days. For EdU assays, cells seeded in 24-well plates (5 × 10^3^ cells/well) were treated with LPS-CM for 2 h after serum starved for 20 h. Scale bars: 100 μm. **b** CCK-8 assays were performed on AML12-NC and AML12-TCTPKD cells. Cells seeded in 96-well plates (1 × 10^3^ cells/well) were incubated with LPS-CM. **c** Representative western blot analysis for the expression of PI3K/AKT pathway-related proteins in AML12-NC, AML12-TCTPKD and the primary hepatocytes obtained from TCTP^+/+^ and TCTP^+/−^ mice. The hepatocytes were treated with LPS-CM for 2 h after serum starved for 20 h. Three independent experiments were carried out. **d**–**f** Representative western blot analysis for the expression of TCTP, AKT and p-AKT^Ser473^ proteins in livers (**d**), the liver weight/body weight ratio (**e**) and Ki67 immunostaining on liver sections (**f**) obtained from TCTP^+/+^ (treated with PBS), TCTP^+/−^ (treated with PBS), and TCTP^+/−^ (treated with IGF-1) mice at day two after PHx. Mice were intraperitoneally injected with PBS or IGF-1 dissolved in PBS at 5 mg/kg/day, for three times a day. Scale bars: 200 μm; *n* = 4. The results were presented as mean ± standard deviation (SD); *P < 0.05; **P < 0.01; ***P < 0.001; ****P < 0.0001 *vs*. control (two-tailed Student’s *t* test for **a**; Two-way ANOVA followed by Tukey’s test for **b**; One-way ANOVA followed by Tukey’s test for (**e**, **f**).

Thereafter, lenti-viruses carrying small guide RNA (sgRNA) or negative control (NC) were transfected into the AML12 hepatocytes, and the efficiency of the gene knockdown was confirmed (Supplementary Figs. S4a). The results demonstrated that TCTP-KD significantly reduced the proliferation of hepatocytes in vitro (Fig. 6a, b). Similar to the in vivo study, mTORC2 and p-AKT^S473^ were inhibited. However, p-PDK1 and p-AKT^T308^ remained unchanged, which differed from the in vivo data. Analogously, the expression of p-RPS6, P27 and cyclinD1 were also altered in TCTP-KD hepatocytes (Fig. 6c). Rescue experiments were performed with IGF-1 (a potent activator of PI3K/AKT signaling^18,23^) in AML12. The IGF-1 treatment upregulated p-AKT^S473^, and effectively restored the proliferation in TCTP-KD hepatocytes (Supplementary Figs. S5-S6). Furthermore, the efficacy of the IGF-1 treatment in restoring LR in TCTP^+/−^ mice was also confirmed (Fig. 6d–f and Supplementary Fig. S7). Taken together, it could be concluded that TCTP facilitates LR by regulating the PI3K/AKT pathway.

### The mTORC2 exerts an important role in the TCTP-elicited activation of PI3K/AKT

**Fig. 7:**
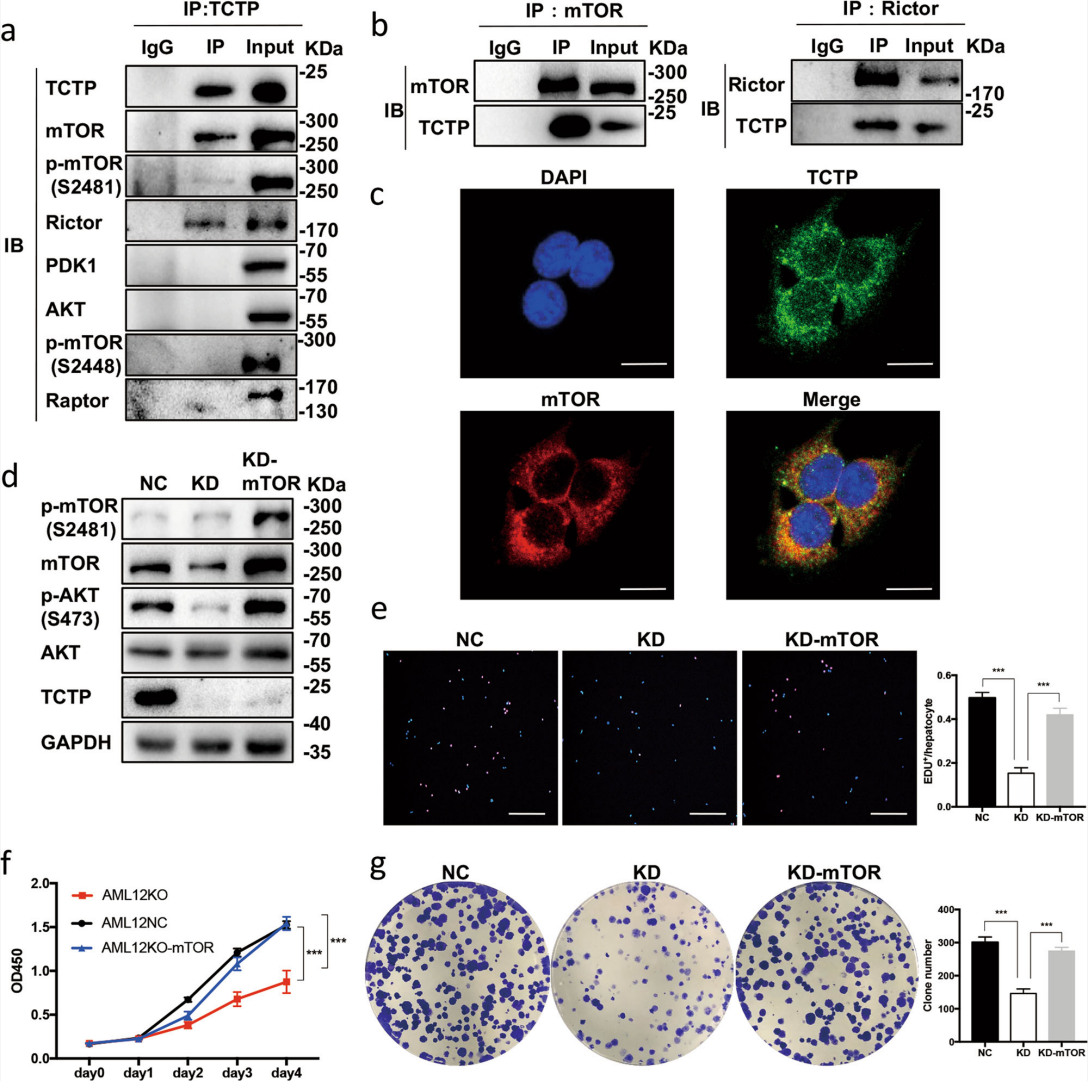
mTORC2 exerts an important role in the TCTP-elicited activation of PI3K/AKT. **a**, **b** Co-IP experiments were performed to detect the interaction among TCTP, mTORC1, mTORC2, PDK and AKT. Protein lysates obtained from AML12 cells treated with the LPS-CM model were immunoprecipitated with the anti-TCTP antibody (**a**), anti-mTOR antibody and anti-Rictor antibody (**b**), and the interactions were subsequently detected by western blot analysis. **c** Representative immunofluorescence images revealed the positional relationship of TCTP and mTOR in AML12 cells treated with the LPS-CM model. Scale bars: 10 μm. **d** Representative western blot analysis for the expression of PI3K/AKT pathway-related proteins in AML12-NC, AML12-TCTPKD and AML12-TCTPKD-mTOR overexpressed cell lines treated with the LPS-CM model. **e**–**g** The EdU assays (**e**), CCK-8 assays (**f**) and colony formation assays (**g**) in AML12-NC, AML12-TCTPKD and AML12-TCTPKD-mTOR cell lines treated with the LPS-CM model. Scale bars: 200 μm. Three independent experiments were carried out. The results were presented as mean ± standard deviation (SD); ***P < 0.001 *vs*. control (Two-way ANOVA followed by Tukey’s test for (**f**); One-way ANOVA followed by Tukey’s test for **e** and **g**).

Immunoprecipitation/mass-spectrum (IP/MS) analysis was used to analyze the products immunoprecipitated with either anti-TCTP antibody or IgG in order to seek some subtracts of TCTP. And the results showed that mTOR, which has a close relationship with PI3K/AKT signaling pathway^24^, can be immunoprecipitated with anti-TCTP antibody, but not IgG (Supplementary Fig. S8a). Then, we confirmed this result by co-immunoprecipitation (co-IP) assays (Fig. 7a, b) and immunofluorescent (IF) staining observed under the confocal microscopy (Fig. 7c and Supplementary Fig. S8b), which consistently revealed that mTOR co-localized TCTP. Given that mTOR is the core subunit of both mTORC1 and mTORC2, and both of the compounds are involved in PI3K/AKT signaling pathway^24^, co-IP assays were utilized to figure out the relationship among TCTP, mTORC1 and mTORC2. The results revealed that RICTOR (a typical subunit of mTORC2), mTOR and p-mTOR^Ser2481^ (active form of mTORC2), except Raptor (a typical subunit of mTORC1) or p-mTOR^Ser2448^ (active form of mTORC1^24^), were immunoprecipitated with the anti-TCTP antibody (Fig. 7a). In turn, TCTP was immunoprecipitated with anti-Rictor antibody (Fig. 7b). Additionally, the mTOR overexpression in TCTP-KD hepatocytes resulted in the reactivation of AKT (Fig. 7d). And the subsequent functional assays confirm the restoration of proliferation in TCTP-KD hepatocytes (Fig. 7e–g). Therefore, it can be concluded that the TCTP/mTORC2/AKT axis facilitates the proliferation of hepatocytes.

### Serum TCTP increased in patients following partial hepatectomy

**Fig. 8:**
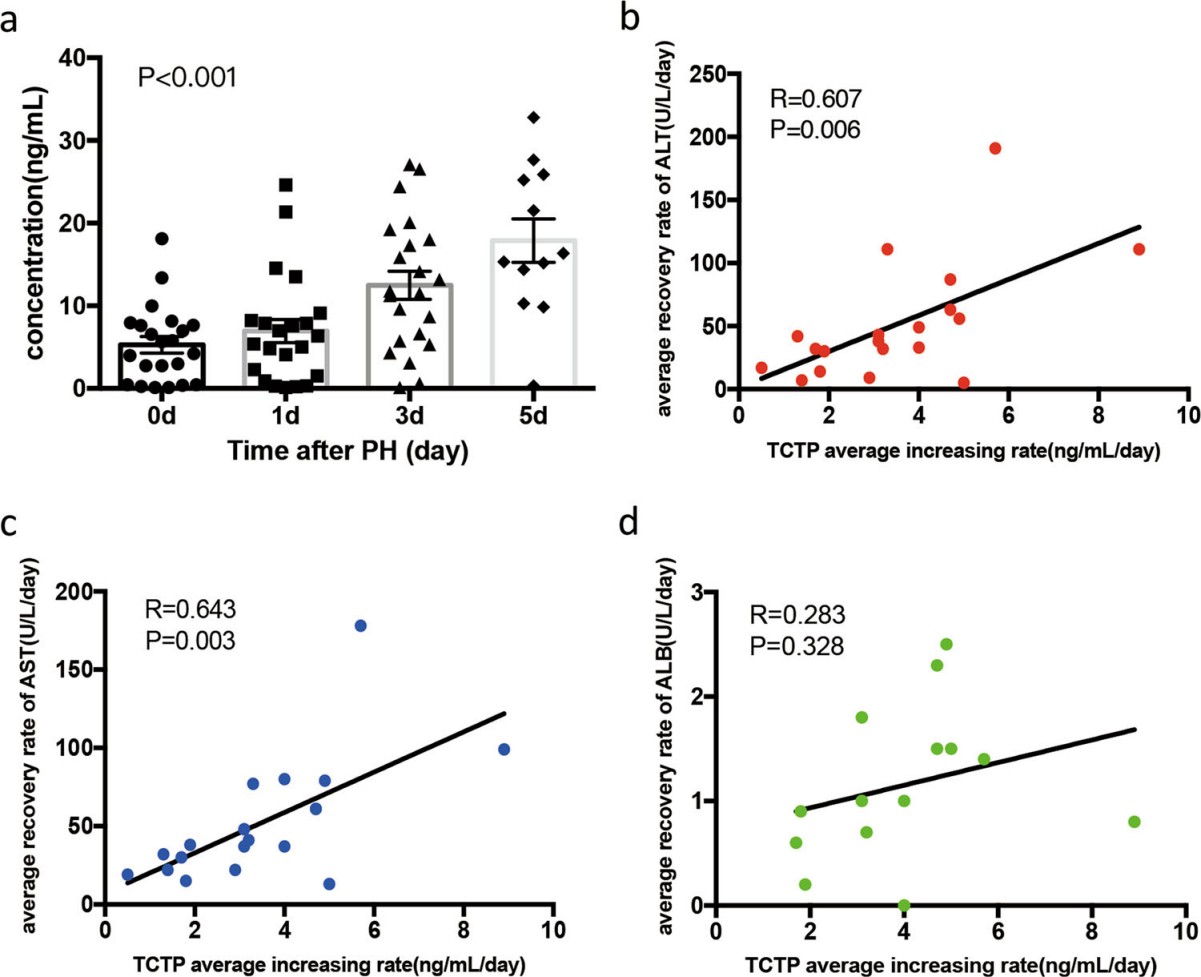
Serum TCTP increased in patients following partial hepatectomy. **a** Serum TCTP in patients undergoing liver resection was detected prior to hepatic surgery, and at day 1, 3 and 5 after liver resection; *n* *=* 22. **b**–**d** A correlation analysis was performed between the average increasing rate of serum TCTP and the average recovery rate of ALT (**b**, *n* *=* 19), AST (**c**, *n* *=* 19) and ALB (**d**, *n* *=* 14), respectively. (Repeated-measures ANOVA for (**a**); Pearson correlation analysis for **b-d**).

Serum samples at different time points before and after PHx were collected from patients (*n* = 22, normal liver function) with hepatic hemangioma (excluding other diseases). Serum TCTP significantly increased in patients after liver resection (Fig. 8a), which was in line with the results obtained from rats^12^. In particular, the relationship between the average increase rate of TCTP and recovery rate of liver function in patients was assessed. Interestingly, it was found that the average decrease rate of ALT and AST positively correlated with the average increase rate of TCTP, without the consideration of patients with no changes in TCTP levels (Fig. 8b, c), and even though no correlation was found between the ALB indicator (excluding patients without ALB changes) and TCTP alteration (Fig. 8d).

## Discussion

LR is mainly regulated by three types of pathways: (i) cytokine, (ii) growth factor, and (iii) metabolic networks. The cytokine network pathway acts at the beginning of LR, allowing quiescent hepatocytes to enter the cell cycle (G0 to G1). Then, the growth factors drive the cell cycle progression by regulating several checkpoints. The role of the metabolic network pathway in the initiation of LR has also been recognized^1^. The present results revealed that TCTP was significantly induced during LR (Fig. 1), which was in accordance with the report of previous studies^11,12^. Furthermore, TCTP-KD restricted LR in mice, and this was accompanied by a delayed cell cycle, abnormal lipid metabolism and lymphocyte infiltration (Figs. 2 and 3).

TCTP has been considered to be able to control the duration of the cell cycle by combining with microtubule and mitotic checkpoints^4^. *AtTCTP* has been proven to act as a positive regulator of mitotic growth by specifically controlling cell cycle progression^10^. Chan et al.^25^ demonstrated that the overexpression of TCTP in hepatocellular carcinoma promoted the degradation of Cdc25C, and subsequently activated Cdk1, leading to a faster mitotic exit. Recently, researchers have revealed that TCTP interacts with CSN4 to promote the G1/S transition in the cell cycle^26^. These present results are analogous with the results of these studies. The mRNA expression of cyclinD1 and cyclinA2, which play important roles in G1/S transition and the S phase of the cell cycle, was depressed, leading to the delayed proliferation of hepatocytes in TCTP^+/−^ mice (Fig. 2e).

Lipid metabolism was identified to take a crucial part in the initiation of LR. Ou-Yang et al.^15^ considered that the absence of Nuclear receptor corepressor 1 (NCoR1) led to the increase in lipid droplets in the liver after PHx, providing energy for hepatocytes to enter the G1 phase of the cell cycle. Meanwhile, the deletion of AKT1 and AKT2 within the liver resulted in a compromised LR, and impaired the accumulation of lipid drops^16^. The excess lipid drops in the liver can impair LR by increasing the sensitivity to apoptosis through TNF-α^27^. In the present study, the formation of lipid droplets in hepatocytes during LR was observed. It has been proven that TCTP plays a role in lipid metabolism. The study conducted by Liu et al.^28^ revealed that genes involved in lipid metabolism were significantly affected by Bombyx mori silkworm TCTP depletion. These lipid drops collected TCTP from mitochondria to maintain the normal function of mitochondria^29^. In the present study, the TCTP-KD reduced the formation of lipid droplets within the liver, and delayed the cell cycle progression in LR (Figs. 2 and 3a).

Increasing evidence has identified the contribution of lymphocytes during LR. The study conducted by Behnke et al.^30^ revealed that B lymphocytes promote LR by regulating the secretion of IL-6 from CD169^+^ macrophages. In addition, T lymphocytes can harmonize with the cellular immune system to promote LR^31^. In the present study, it was discovered for the first time that lymphocytes infiltrated in flocks within the liver of TCTP^+/−^ mice during LR (Fig. 3b). Combined with the results of the RNA sequencing (Fig. 4b), it can be deduced that the relationship between TCTP and immune response might contribute to LR, which needs to be further investigated.

The mTOR is a serine/threonine protein kinase that belongs to the PI3K-related kinase family, and forms two complexes (mTORC1 and mTORC2)^24^. Activated mTORC2 can phosphorylate AKT^Ser473^, leading to the full activation of AKT^18^, which subsequently promotes mTORC1 activation and cell proliferation^13,16,24^. The interaction between TCTP and mTOR signaling pathway is complicated. Previously, *dTCTP* was found to interact with *dRheb* (a key factor in TOR signaling), facilitating its function as a guanine nucleotide exchange factor^8^. Furthermore, the positive feedback loop between TCTP and mTOR contributes to the development of NF1-associated tumors^32^. The results of the present study indicate that TCTP regulates the level of mTORC2, which subsequently activates the AKT signaling in LR.

Three possible explanations were proposed for the TCTP-mTORC2 interaction: (i) TCTP facilitates the mTORC2 synthesis; (ii) TCTP promotes the mTORC2 phosphorylation; (iii) TCTP inhibits the mTORC2 degradation. Firstly, it was not considered that point (i) is correct, because the Co-IP assay illustrated that TCTP interacted with the mTORC2 protein (Fig. 7a, b), and no study has reported that TCTP can promote the transcription of mTOR. Meanwhile, the results suggest that the TCTP-KD downregulated not only the level of mTOR, but also the level of p-mTOR^Ser2481^ (Fig. 6c). Hence, point (ii) was excluded (If TCTP promotes the phosphorylation and activation of mTORC2, the TCTP-KD would not influence the level of mTOR). Lastly, since the results revealed that the mTOR overexpression restored the p-AKT^Ser473^ level and the cell proliferation (Fig. 7d–g), it can be deduced that the mTOR protein level is the key to TCTP’s effect on LR. Hence, TCTP, which seems unlikely to promote the synthesis of mTOR, might facilitate LR through inhibiting the degradation of mTORC2. However, further studies need to be conducted to confirm this hypothesis.

Furthermore, it was also considered that the inactive TCTP/mTORC2/AKT axis accounted for the delayed cell cycle and impaired adipogenesis in TCTP^+/−^ mice during LR, since: (i) both mTORC2 and AKT exerts considerable influence on the cell cycle and lipid metabolism;^13,16,24,33,34^ (ii) the treatment with IGF-1 restored the phosphorylation of AKT and lipid drops accumulation in TCTP^+/−^ mice (Fig. 6d–f).

Although the western blot results revealed that the TCTP-KD hampered the phosphorylation of RPS6 (Fig. 6c), a downstream substrate of mTORC1, no interactions between TCTP and mTORC1 were detected in the Co-IP assays (Fig. 7a). Since mTORC1 is one of the effectors of the PI3K/AKT pathway, we believe that the mTORC1 suppression in TCTP-KD results from the lack of activation of mTORC2 by TCTP.

Since extracellular TCTP was reported to exert cytokine-like activities to stimulate the histamine release from basophils, and facilitate the progression of colorectal cancer cells^4,35,36^, the serum level of TCTP secreted in patients following PHx was investigated. The present analysis revealed that the accumulation of TCTP in serum was correlated to the recovery of liver function, hinting the potential role of TCTP as a biomarker of LR (Fig. 8).

In conclusion, the interaction between TCTP and mTORC2 was demonstrated, and it was unraveled that the TCTP/mTORC2/AKT signal pathway exerts a crucial role in LR. In addition, a correlation between serum TCTP and the indicators of LR in humans was established for the first time in the present study.

## Materials and methods

### Mice and surgical procedure

Gene targeting technology was used to generate the TCTP^+/−^ mice. Briefly, a targeting vector that contained a series of functional sites was linearized and electroporated into embryonic stem cells derived from the C57BL/6 background, and the targeted embryonic stem cells were injected into the blastocysts of Balb/c mice. Then, the chimeras were mated with C57BL/6 mice, and the *Tpt1* mutation was transmitted to the germ line (Supplementary Fig. S1a). Afterwards, the genomic DNA was extracted from the mouse tail samples for genotyping (Supplementary Fig. S1b). The primer sequences used for mice genotyping are presented in Supplementary Figure S1c. Mice were raised in a specific pathogen free (SPF) facility, and were given free access to water and rodent diet, with a 12-h light-dark cycle. Young adult (8–12 weeks) male mice were randomly chosen for the experiments using a table of random numbers. The sample size is estimated to effectively detect a significant difference among the groups. The investigator was blinded to the group allocation. Mice were subjected to PHx or sham operation.The rat 70% PHx method of Higgins and Johnson^37^ was modified to remove the left and median lobes of the liver. The sham operation consisted of laparotomy without liver resection. After the PHx or sham operation, these wild-type (WT) mice, TCTP^+/+^ mice and TCTP^+/−^ mice were sacrificed at different time points to collect the blood and tissue. All surgeries were carried out under isoflurane anesthesia. The flow of 2% isoflurane was set at a rate of 0.5 L/min. For the rescue experiments, mice were intraperitoneally injected with PI3K/AKT stimulator IGF-1 (5 mg/kg/day, three times per day; R&D System) dissolved in phosphate-buffered saline (PBS) or PBS as control. The liver/body weight was calculated using the following formula: weight of non-removed lobes / total body weight of mice. The study was approved by the Ethics Committee of Xijing Hospital.

### RNA isolation and quantitative polymerase chain reaction

Trizol (Invitrogen, Life Technologies, CA, USA) reagent was used to isolate the total RNA from the liver or hepatocytes, according to the manufacturer’s protocol. The total RNA was reverse-transcribed using a reverse transcription kit (Invitrogen), according to manufacturer’s instructions. Then, the cDNA was analyzed by real-time polymerase chain reaction (RT-PCR) using the SYBR Green PCR master mix (Takara, Japan) in a two-step reaction. Each reaction was repeated in triplicate wells. The target specific primer sequences for the RT-PCR are presented in Supplementary Table 1. The expression of the gene was calculated using the 2 ^- (△△Ct)^ method.

### Western blot analysis

Protein lysates were prepared in RIPA buffer (50 mM of Tris pH 7.4, 150 mM of NaCl, 1% TritonX-100, 1% sodium deoxycholate, 0.1% SDS, 2 mM of sodium pyrophosphate, and 1 mM EDTA; Beyotime, China) supplemented with a protease inhibitor cocktail (Thermo, Rockford, IL, USA). Then, the protein lysates were separated by 8% or 10% sodium dodecyl sulfate-polyacrylamide gel electrophoresis (SDS-PAGE), and transferred onto polyvinylidene fluoride (PVDF) membranes. Afterwards, the membranes were blocked for 60 min at room temperature, incubated with the primary antibodies (Supplementary Table 2) at 4 °C overnight, and incubated with the appropriate secondary antibodies for 1 h at room temperature. The bands were probed using the ChemiDoc MP Imager (Bio-Rad).

### Immunohistochemistry (IHC) assay

The regenerating lobes used for the hematoxylin and eosin (H&E) staining and immunostaining were fixed overnight in 4% paraformaldehyde, embedded in paraffin, and cut into 5-μm-thick sections for the IHC assay. The primary antibodies are listed in Supplementary Table 2. In order to count the Ki67-positive hepatocytes, five fields were randomly chosen per section after the immunostaining. The percentage of Ki67-positive hepatocytes was calculated to quantify the hepatocyte proliferation. In order to identify the cytoplasmic lipid vacuoles, the regenerating lobes were fixed in 4% paraformaldehyde for 6 h, cryoprotected overnight in 30% sucrose solution, and embedded in optimal cutting temperature medium for Oil Red O staining (Sigma-Aldrich).

### Enzyme-linked immunosorbent assay and biochemistry

From November 2018 to June 2019, 22 patients with hepatic hemangioma undergoing hepatic resection at the Department of Hepatobiliary Surgery, Xijing Hospital of the Fourth Military Medical University were enrolled to the present study. All patients provided a signed written informed consent for the use of clinical specimens for medical research. Human serum samples were collected on the day before the surgery, and at postoperative 24, 72, and 120 h. After centrifugation at 1000*g*, the serum was extracted and stored at −20 °C. Human TCTP was detected using a human translationally-controlled tumor protein ELISA kit from SAB (EK3951; Signalway Antibody), according to manufacturer’s instructions. The present study was approved by the Ethics Review Committee of Xijing Hospital. The serum ALT and AST levels of mice were analyzed using a Chemistry Analyzer (AU400, Olympus), according to manufacturer’s instructions.

### RNA sequencing

The total RNA was isolated from the primary hepatocytes of TCTP^+/+^ mice (*n* = 3) and TCTP^+/−^ mice (*n* = 3). The RNA samples were analyzed by RNA sequencing (BGI, Shenzhen, China) through the BGISEQ-500 platform. Genes that were significantly and differentially expressed between TCTP^+/+^ and TCTP^+/−^ mice were selected based on a fold change of >2.0 and a *P*-value of < 0.05, and subsequently analyzed by KEGG pathway enrichment analysis. The results of the RNA sequencing was submitted to the Sequence Read Archive (SRA) database (Accession number: PRJNA553638).

### Establishment of the in vitro LR model

Aydemir, T. B.’s protocol^22^ was modified to prepare the macrophage-conditioned medium. Briefly, RAW264.7 mouse macrophages were seeded into 6-well plates at a density of 2 × 10^5^ cells/well, and incubated with Dulbecco’s Modified Eagle’s Medium (DMEM), containing 10% inactivated (56 °C, 30 min) fetal bovine serum (FBS) (Gibco, USA). After 48 h, cells were treated with either 20 μg/ml of lipopolysaccharide (LPS; Sigma-Aldrich, Germany), or phosphate buffer solution (PBS) for 2 h. Then, the supernatants were collected from the LPS-treated and PBS-treated macrophages, and were named as LPS-CM and CM, respectively. In order to establish the in vitro LR model, the hepatocytes were treated with LPS-CM after serum starved for 20 h. After 2 h, the protein or total RNA was extracted, and the following experiments were conducted.

### Lentivirus transfection and stable cell clone establishment

The negative control lenti-virus and lenti-virus loading CRISPR/Cas9 system targeting genomic TCTP sequences were purchased from GENECHEM (Shanghai, China). The sgRNA sequences were as follows: sgRNA1 AGATCCGGGAGATCGCGGAC; sgRNA2: AGATCCGGGAGATCGCGGAC; sgRNA3: AGATCCGGGAGATCGCGGAC. After testing the efficiency of these three sgRNAs, sgRNA1 were selected for the subsequent experiments. The negative control lenti-virus and lenti-virus loading a plasmid, which carries the mTOR gene, were purchased from GeneCopoeia (Maryland, USA; Cat. no. Mm31144). Cells were planted in 6-well plates and cultured overnight. The lenti-virus was infected into AML12 or AML12-TCTPKD cells at 100 multiplicity of infection (MOI) with a transduction-enhancing solution. After 12 h, the medium was replaced with the complete medium.

### Cell lines and culture

Mouse macrophages RAW264.7 and mouse normal hepatic cell line AML12 were kindly provided by the Stem Cell Bank, Chinese Academy of Sciences (Shanghai, China). RAW264.7 was cultured in DMEM (Hyclone) supplemented with 10% inactivated FBS. Then, AML12 was maintained in DMEM/F-12 (Invitrogen, Cat. no. 11330-032) containing 10% FBS (Gibco), 40 ng/mL of dexamethasone (Sigma), and ITS Liquid Media Supplement (Sigma, I3146). All cells were cultured in a humidified atmosphere with 5% CO_2_ at 37 °C. Mycoplasma Detection Kit (Solarbio, China) was used to detect mycoplasma contamination.

### Isolation and culture of primary hepatocytes

The primary hepatocytes were isolated according to the protocol of Duan et al.^38^. Under isoflurane anesthesia, mice were perfused with Hank’s buffer without Ca^2+^ from the portal vein, followed by Hank’s buffer with Ca^2+^ and Mg^2+^, containing 0.2 mg/mL of collagenase IV (Sigma-Aldrich). Then, the liver was grounded using a tissue homogenizer in a perfusion buffer containing 0.2 mg/mL of collagenase IV and 10 μg/mL of DNase I (Roche, Basel, Switzerland). Afterwards, the slurry was filtered using a 70-μm cell mesh to obtain a single-cell suspension. Centrifugation at 50*g* was preformed for 3 min to precipitate the hepatocytes. The primary hepatocytes were maintained in hepatocyte medium (ScienCell, USA; Cat. no. 5201) for the subsequent assays.

### Cell counting kit-8 (CCK-8) assay

CCK-8 (Beyotime, China) was used to assess the cell proliferation viability. Cells were seeded into 96-well plates at a density of 1 × 10^3^ cells/well with 100 μl of LPS-CM. Then, 10 μl of CCK-8 reagent was added to each well at the indicated time points (6, 24, 48, 72 and 96 h). Afterwards, the plates were incubated in the dark at 37 °C for 1 h, and read using a microplate reader (Bio-Tek Elx 800; Bio-Tek Instruments, Winooski, VT, USA) at 450 nm. For the rescue experiments, cells were treated with LPS-CM containing PI3K/AKT stimulator IGF-1 (100 ng/mL, R&D System) or PBS as the control. The experiments were conducted in triplicate.

### Colony formation assay

Cells were seeded into 6-well plates at a density of (0.5–1.0) × 10^3^ cells/well. After culturing with LPS-CM for 1–2 weeks, the colonies on the plates were fixed in 4% paraformaldehyde and stained with 1% crystal violet, and the number of colonies were counted to evaluate the cell proliferation. For the rescue experiments, cells were treated with LPS-CM containing PI3K/AKT stimulator IGF-1 (100 ng/mL, R&D System) or PBS as the control. The experiments were performed in triplicate.

### The 5-ethynyl-2′-deoxyuridine (EdU) incorporation assay

Cell proliferation was measured through the incorporation of EdU during the DNA synthesis using a Cell-Light™ EdU Apollo®567 In Vitro Imaging Kit (Ribobio, Guangzhou, China), according to manufacturer’s instructions. Cells were seeded into 24-well plates at a density of (0.5–1.0) × 10^4^ cells/well, and incubated overnight. Then, these cells were treated with LPS-CM for 2 h after serum starved for 20 h. Thereafter, these cells were incubated with EdU for 2 h at 37 °C, and fixed in 4% paraformaldehyde. After permeabilization with 0.5% Triton X-100 in PBS, these cells were reacted with the 1 × Apollo reaction cocktail for 30 min. Subsequently, the nuclei were stained with Hoechst for 15 min, and the EdU positive cells were captured and quantified by fluorescence microscopy. For the rescue experiments, cells were treated with LPS-CM containing IGF-1 (100 ng/mL, R&D System) or PBS as the control. All studies were conducted in triplicate.

### Immunofluorescence (IF)

Cells were seeded into 24-well plates or slides at a density of 2 × 10^4^ cells/well, and incubated overnight. Then, these cells were fixed with 4% paraformaldehyde solution for 15 min, permeabilized with 0.1% Triton X, and blocked with bovine serum albumin (BSA) for 1 h. Subsequently, these cells were washed with PBS and treated with the secondary antibodies. Afterwards, the images were collected under a fluorescence microscope or confocal laser scanning microscope. The primary antibodies are presented in Supplementary Table 2. For the rescue experiments, cells were pretreated with IGF-1 (100 ng/mL, R&D System) or PBS as the control. All studies were conducted in triplicate.

### Co-immunoprecipitation (Co-IP) and mass-spectrum (MS) assays

The total protein was extracted from AML12 cells treated with LPS-CM using a modified RIPA buffer (P0013D; Beyotime), and immunoprecipitated with the primary antibody (presented in Supplementary Table 2) against TCTP, mTOR and RICTOR overnight at 4 °C. Subsequently, 20–40 μL of fully suspended protein A + G agarose (P2006, Beyotime) was added and incubated for 3 h. The complexes that bound to the protein A + G-agarose conjugate were resolved on the SDS-PAGE loading buffer, and subjected to western blotting. The IP products (immunoprecipitated with either anti-TCTP antibody or IgG) were analyzed by MS. MS was conducted by equipments of Q Exactive (Thermo Fisher) and Easy-nLC 1000(Thermo Fisher). The data of MS were analyzed by MASCOT Software. MS assyas were supported by Shanghai Applied Protein Technology (Shanghai, China).

### Statistical analysis

The statistical analysis was performed using SPSS 25.0 (SPSS, Chicago, IL, USA) and Prism 7.0 (GraphPad Software, La Jolla, CA, USA). Data were presented as mean ± standard deviation (SD), unless otherwise stated. Two-sided student’s *t* test or one-way analysis of variance (ANOVA) were used to compare two or three different groups of homoscedasticity and normally distributed data (assessed by Shapiro–Wilk test). Two-way ANOVA (followed by Tukey’s post hoc test for multiple comparisons) was used to compare two preselected groups that involved more than one time-point. ANOVA for repeated measurement was used to measure the circulating TCTP levels in patients undergoing liver resection. Pearson correlation analysis was used to measure the correlation between serum TCTP and some liver function indicators. The statistical tests above are justified as appropriate. *P* < 0.05 was considered statistically significant.

## Supplementary information

Supplemental Material

supplementary figure 1

supplementary figure 2

supplementary figure 3

supplementary figure 4

supplementary figure 5

supplementary figure 6

supplementary figure 7

supplementary figure 8

Dataset 1

Dataset 2

Dataset 3

Dataset 4
