# Correction: Translationally controlled tumor protein promotes liver regeneration by activating mTORC2/AKT signaling

**DOI:** 10.1038/s41419-020-2311-9

**Published:** 2020-02-11

**Authors:** Zhibin Lin, Xuan Zhang, Jianlin Wang, Wei Liu, Qi Liu, Yuchen Ye, Bin Dai, Dongnan Guo, Pengcheng Zhang, Peijun Yang, Ruohan Zhang, Lin Wang, Kefeng Dou

**Affiliations:** Department of Hepatobiliary Surgery, Xijing Hospital, The Fourth Military Medical University, Xi’an, China

**Keywords:** Cell biology, Molecular biology

**Correction to**: **Cell Death & Disease**

10.1038/s41419-020-2231-8 published online 23 January 2020

Reference 26 reads “Hua, J. et al. TCTP and CSN4 control cell cycle progression and development by regulating CULLIN1 neddylation in plants and animals. PLoS Genet. 15, e1007899 (2019).” However, it should read “Betsch, L. et al. TCTP and CSN4 control cell cycle progression and development by regulating CULLIN1 neddylation in plants and animals. PLoS Genet. 15, e1007899 (2019).”

This has been corrected in the PDF and HTML versions.

