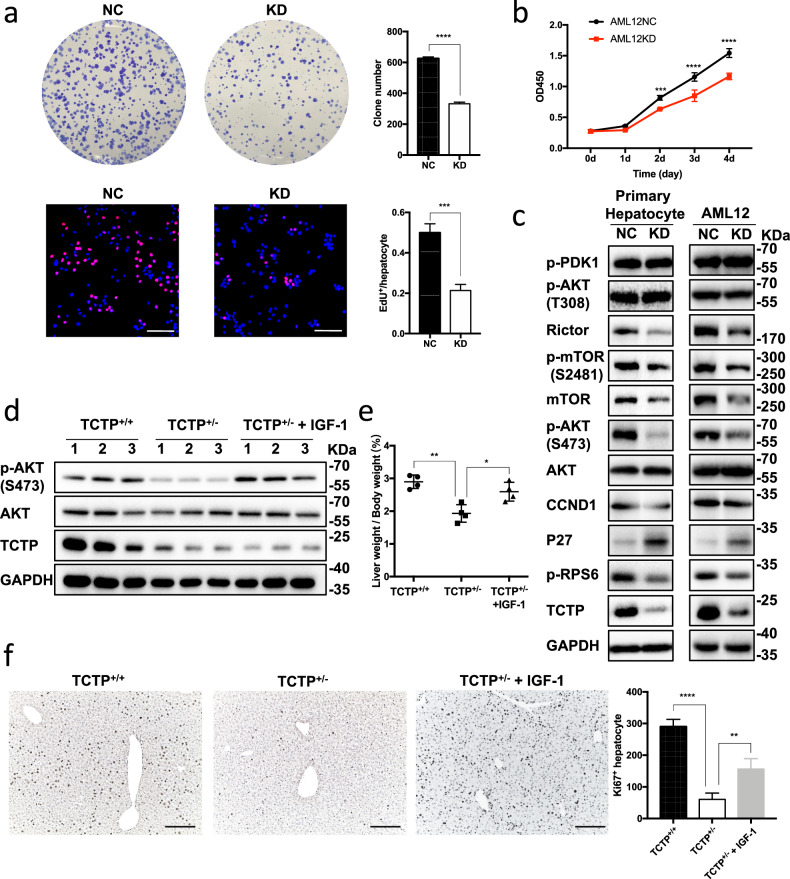# Correction: Translationally controlled tumor protein promotes liver regeneration by activating mTORC2/AKT signaling

**DOI:** 10.1038/s41419-024-06590-0

**Published:** 2024-04-04

**Authors:** Zhibin Lin, Xuan Zhang, Jianlin Wang, Wei Liu, Qi Liu, Yuchen Ye, Bin Dai, Dongnan Guo, Pengcheng Zhang, Peijun Yang, Ruohan Zhang, Lin Wang, Kefeng Dou

**Affiliations:** Department of Hepatobiliary Surgery, Xijing Hospital, The Fourth Military Medical University, Xi’an, China

Correction to: *Cell Death and Disease* 10.1038/s41419-020-2231-8, published online 23 January 2020

The original version of this article contains a mistake in Fig. 6f. When integrating the figures, one field of the “2d TCTP^+/-^” liver section in Fig. 2c was wrongly selected for the “TCTP^+/-^ +IGF-1” group in Fig. 6f, since liver tissues from both of the groups were obtained at day 2 after partial hepatectomy. Besides, the authors zoomed in and cut the images in Fig. 2c, otherwise they would too large to put in the Figure 2. And the authors adjusted the colour intensity of the images to make them accordant in the corresponding panels (since the authors only counted the positive hepatocytes, colour intensity adjustment had no impact on the results). Hence, the two overlapping images are scaled differently, and have different colour intensity. To rectify this mistake, the Ki67 staining image of the “TCTP^+/-^ +IGF-1” group in Fig. 6f has been replaced. The corrected figure can be found below. The authors apologize for this mistake. There is no impact on the final conclusions.