# Supplementary material for: Translationally controlled tumor protein promotes liver regeneration by activating mTORC2/AKT signaling
Source: Cell Death Dis. 2020 Jan 23;11(1):58. doi: 10.1038/s41419-020-2231-8 (PMC6978394; doi:10.1038/s41419-020-2231-8)
Supplement: Supplementary file 1 — Supplemental Material [file 41419_2020_2231_MOESM1_ESM.doc]

**Supplemental material**

**Supporting Table S1.** Primers used in the present study

| Gene | Forward primer | Reverse primer |
| --- | --- | --- |
| TCTP | GAGCTGCAGAGCAGATTAAG | CCATCTTCACGGTAGTCCAG |
| GAPDH | AGGTCGGTGTGAACGGATTTG | TGTAGACCATGTAGTTGAGGTCA |
| IL-6 | TAGTCCTTCCTACCCCAATTTCC | TTGGTCCTTAGCCACTCCTTC |
| TNF-α | ACGTCGTAGCAAACCACCAA | ATCGGCTGGCACCACTAGTT |
| HGF | ATGTGGGGGACCAAACTTCTG | GGATGGCGACATGAAGCAG |
| CCND1 | GCGTACCCTGACACCAATCTC | CTCCTCTTCGCACTTCTGCTC |
| CCNA2 | AAGAGAATGTCAACCCCGAAAAA | ACCCGTCGAGTCTTGAGCTT |
| P21 | CCTGGTGATGTCCGACCTG | CCATGAGCGCATCGCAATC |
| P27 | TCAAACGTGAGAGTGTCTAACG | CCGGGCCGAAGAGATTTCTG |
| Ki67 | ATCATTGACCGCTCCTTTAGGT | GCTCGCCTTGATGGTTCCT |
| PCNA | TTTGAGGCACGCCTGATCC | GGAGACGTGAGACGAGTCCAT |
| mTOR | ACCGGCACACATTTGAAGAAG | CTCGTTGAGGATCAGCAAGG |

**Supporting Table S2.** Antibodies used in the present study

| Antigens | Manufacturers | Application |  |
| --- | --- | --- | --- |
| TCTP | ab133568; Abcam | 1:10000 for WB |  |
|  |  | 1:10 for IP |  |
|  |  | 1:250 for IHC-P |  |
| TCTP | 66713-1-Ig; Proteintech | 1:50 for IF |  |
| GAPDH | #5174; Cell Signaling Technology | 1:4000 for WB |  |
| AKT | #4685; Cell Signaling Technology | 1:1000 for WB |  |
| P-AKT(S473) | #4060; Cell Signaling Technology | 1:2000 for WB |  |
|  |  | 1:250 for IHC-P |  |
| P-AKT(T308) | #13038; Cell Signaling Technology | 1:1000 for WB |  |
| mTOR | #2983; Cell Signaling Technology | 1:1000 for WB |  |
|  |  | 1:250 for IHC-P |  |
|  |  | 1:200 for IF |  |
| mTOR | #2972; Cell Signaling Technology | 1:10 for IP |  |
| P-mTOR(S2481)  P-mTOR(S2448) | #2974; Cell Signaling Technology  #5536; Cell Signaling Technology | 1:1000 for WB  1:1000 for WB |  |
| Rictor  Raptor  p-RPS6(S235/236)  P27  CCND1 | #2114; Cell Signaling Technology  ab70374; Abcam  #2208; Cell Signaling Technology  #4858; Cell Signaling Technology  25614-1-AP; Proteintech  60186-1-Ig; Proteintech | 1:1000 for WB  10 µg/mg for IP  1:1000 for WB  1:1000 for WB  1:1000 for WB  1:5000 for WB | |
| P-PDK1(S241) | #3438; Cell Signaling Technology | 1:1000 for WB |  |
| Ki67 | ab15580; Abcam | 1:200 for IHC-P |  |
| PTEN | #9552; Cell Signaling Technology | 1:1000 for WB |  |
| P-PTEN  CD3  CD19 | #9554; Cell Signaling Technology  GB13014; Servicebio  GB11061-1; Servicebio | 1:1000 for WB  1:3000 for IF  1:5000 for IF |  |

**Figure legends**

**Supplementary Figure 1** **Generation and** **identification of TCTP^+/-^ mice.** **(a)** The schematic of the construct to knockdown the TCTP gene (*Tpt1*) using gene targeting technology. This targeting strategy incorporates an FRT site, En2 SA-IRES-eGFP-polyA sequence (this sequence could inhibit the transcription of downstream *Tpt1* sequence) and a loxP site, followed by a neomycin resistance cassette, a second FRT site and a second loxP site between exons 2 and 3, while allowing for the floxing of exons 3 and 4 for subsequent conditional KO studies. **(b)** Agarose gel electrophoresis (AGE) was conducted for the genotyping of samples from TCTP^+/+^ and TCTP^+/-^ transgenic mice. The size of gene products of the homozygotes is 289 bp, while the size of gene products of the heterozygotes is 289 bp and 341 bp. **(c)** The sequence of primers used for the PCR, which amplifies the samples from TCTP^+/+^ and TCTP^+/-^ transgenic mice for the genotyping.

**Supplementary Figure 2 Abnormal immune response in TCTP^+/-^ mice during LR.** Representative immunofluorescence staining of CD3 (green) and CD19 (red) on liver sections obtained from the livers of the TCTP^+/-^ mice at day two after PHx. Scale bars: 50 μm.

**Supplementary Figure 3** **TCTP activates PI3K/AKT signaling during LR.** **(a)** Representative immunohistochemistry staining of TCTP, mTOR and p-AKT^Ser473^ (brown) on liver sections obtained from the livers of TCTP^+/+^ and TCTP^+/-^ mice at day two after PHx; scale bars: 100 μm (upper) and 50 μm (lower); *n*=5. **(b)** Representative western blot analysis for the proteins expression of PTEN and p-PTEN in the livers of three randomly chosen TCTP^+/+^ mice and three randomly chosen TCTP^+/-^ mice at day two after PHx. The blots of TCTP and GAPDH were the same as those in Figure 4e.

**Supplementary Figure 4** **The construction of cell lines mentioned in the present study.** **(a)** Negative control lenti-virus and lenti-virus loading sgRNA were transfected into the AML12 cell line. The efficiency of TCTP-KD was confirmed by RT-PCR. The results are represented as mean ± standard deviation (SD); ***P*<0.01 *vs.* control (two-tailed Student’s *t*-test). **(b)** The negative control lenti-virus and lenti-virus loading a plasmid that carries mTOR gene were transfected into the AML12-TCTP-KD cell lines. The efficiency of mTOR-overexpression was confirmed by RT-PCR. Three independent experiments were carried out. The gray images revealed the microscopic features of AML12 cells transfected with the TCTP-KD-virus (**a**) and AML12-TCTP-KD cells transfected with the mTOR-overexpress-virus (**b**). These two kinds of viruses were both labeled with green fluorescent protein (GFP). The green images show the expression of GFP in each cell. Scale bars: 100 μm.

**Supplementary Figure 5** **The impaired proliferation of AML12-TCTPKD cells was rescued by IGF-1**. **(a)** Representative western blot analysis for the protein expression of TCTP, AKT and p-AKT^Ser473^ in AML12-NC (treated with PBS), AML12-TCTPKD (treated with PBS) and AML12-TCTPKD (treated with IGF-1, 100 ng/mL) cells. **(b-d)** CCK-8 assays (**b**), EdU assays (**c**), and colony formation assays (**d**) was performed on AML12-NC (treated with PBS), AML12-TCTPKD (treated with PBS) and AML12-TCTPKD (treated with IGF-1, 100 ng/mL) cells. For the western blot analysis and EdU assays, cells were treated with PBS or LPS-CM for two hours after the serum was starved for 20 hours. For the CCK-8 assays, cells were treated with PBS or LPS-CM for four days. For the colony formation assays, cells were treated with PBS or LPS-CM for 12 days. Scale bars: 100 μm. Three independent experiments were carried out. The results are represented as mean ± standard deviation (SD); **P*<0.05; ***P*<0.01; ****P*<0.001 *vs.* control (Two-way ANOVA, followed by Tukey’s test for **b**; One-way ANOVA, followed by Tukey’s test for **c** and **d**).

**Supplementary Figure 6** **The impaired proliferation of primary hepatocytes obtained from TCTP^+/-^ mice was rescued by IGF-1.** **(a-c)** Representative western blot analysis for the protein expression of TCTP, AKT and p-AKT^Ser473^ (**a**), the RT-PCR analysis for the mRNA expression of PCNA and Ki67 (**b**), and the immunofluorescence staining of Ki67 (**c**) in primary hepatocytes obtained from TCTP^+/+^ mice, primary hepatocytes obtained from TCTP^+/-^ mice (treated with PBS), and primary hepatocytes obtained from TCTP^+/-^ mice (treated with IGF-1, 100 ng/mL). For all the experiments above, cells were treated with PBS or LPS-CM for two hours after serum starved for 20 hours. Scale bars: 200 μm. Three independent experiments were carried out. The results are represented as mean ± standard deviation (SD); **P*<0.05; ***P*<0.01 *vs.* control (One-way ANOVA, followed by Tukey’s test).

**Supplementary Figure 7 The impaired lipid accumulation and liver function of TCTP^+/-^ mice after PHx was rescued by IGF-1**. **(a)** Representative histological panels of the Oil Red O staining in liver sections (red) obtained from TCTP^+/+^ (treated with PBS), TCTP^+/-^ (treated with PBS), and TCTP^+/-^ (treated with IGF-1) mice at day two after PHx. **(b-c)** The serum ALT (**b**) and AST (**c**) level of TCTP^+/+^ (treated with PBS), TCTP^+/-^ (treated with PBS), and TCTP^+/-^ (treated with IGF-1) mice at day two after PHx. Mice were intraperitoneally injected with PBS or IGF-1 dissolved in PBS at 5 mg/kg/day, for three times a day. Scale bars: 100 μm; *n*=4. The results are represented as mean ± standard deviation (SD); **P*<0.05 *vs.* control (One-way ANOVA, followed by Tukey’s test).

**Supplementary Figure 8** **TCTP interacts mTOR.** **(a)** Immunoprecipitation/mass spectrum (IP/MS) analysis was performed to determine the proteins that interact TCTP. mTOR was revealed as one of the interacting protein. **(b)** The representative immunofluorescence images show that TCTP co-localized mTOR in primary hepatocytes. Cells were treated with LPS-CM for two hours after serum starved for 20 hours. scale bars: 10 μm. Three independent experiments were carried out.
