# Supplementary figures and images for: Translationally controlled tumor protein promotes liver regeneration by activating mTORC2/AKT signaling
Source: Cell Death Dis. 2020 Jan 23;11(1):58. doi: 10.1038/s41419-020-2231-8 (PMC6978394; doi:10.1038/s41419-020-2231-8)

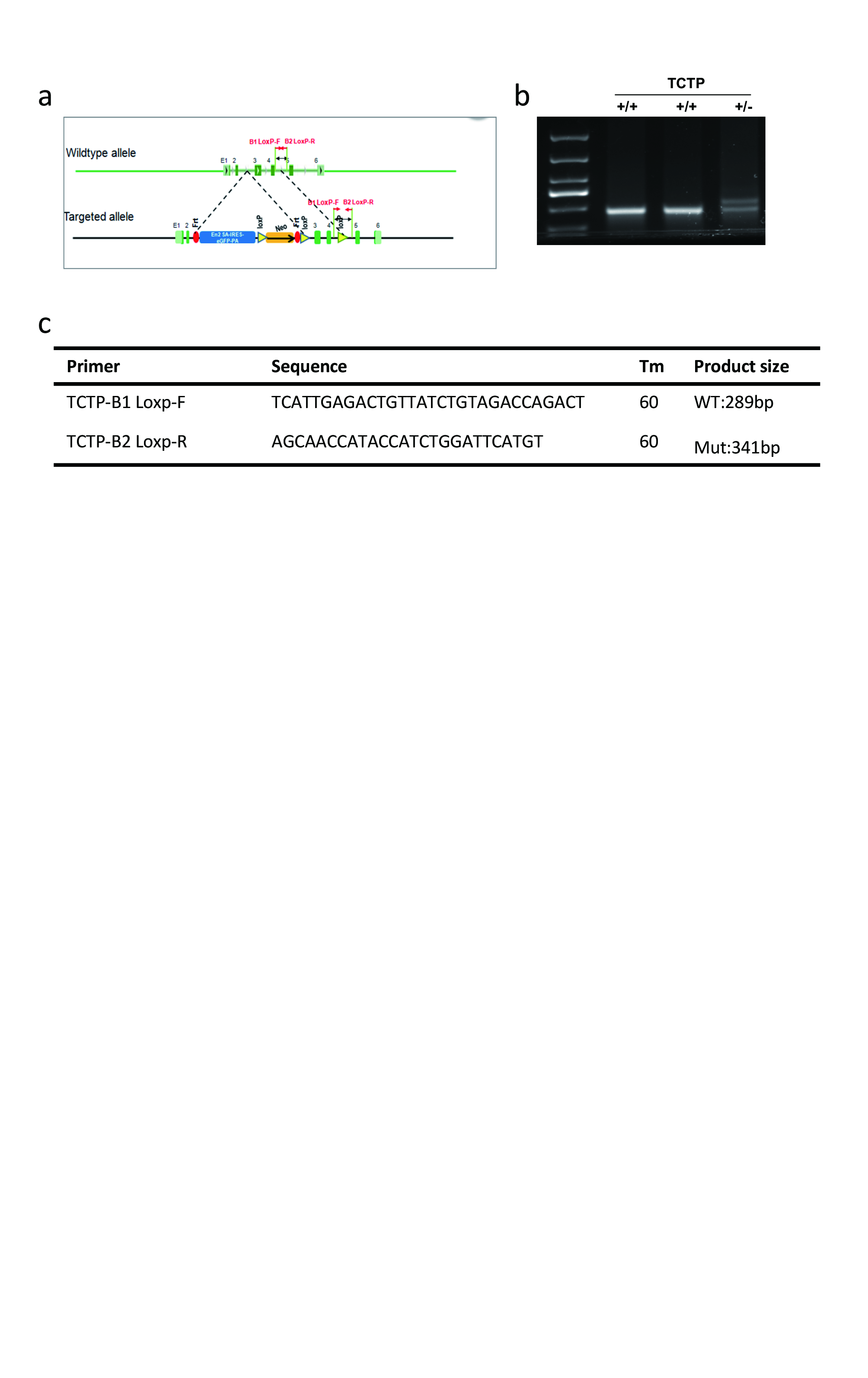

Supplement: Supplementary file 2 — supplementary figure 1 [file 41419_2020_2231_MOESM2_ESM.tif]

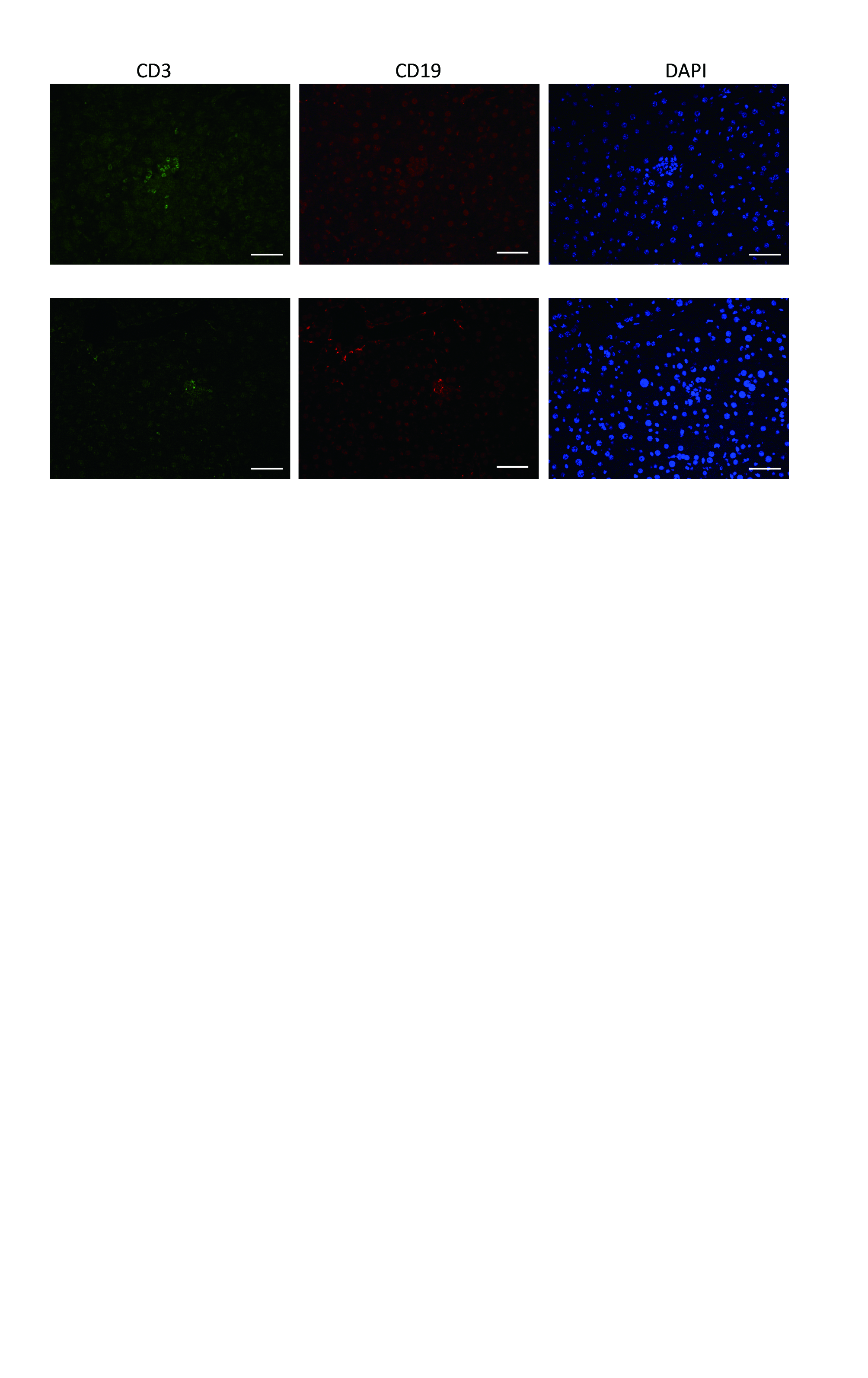

Supplement: Supplementary file 3 — supplementary figure 2 [file 41419_2020_2231_MOESM3_ESM.tif]

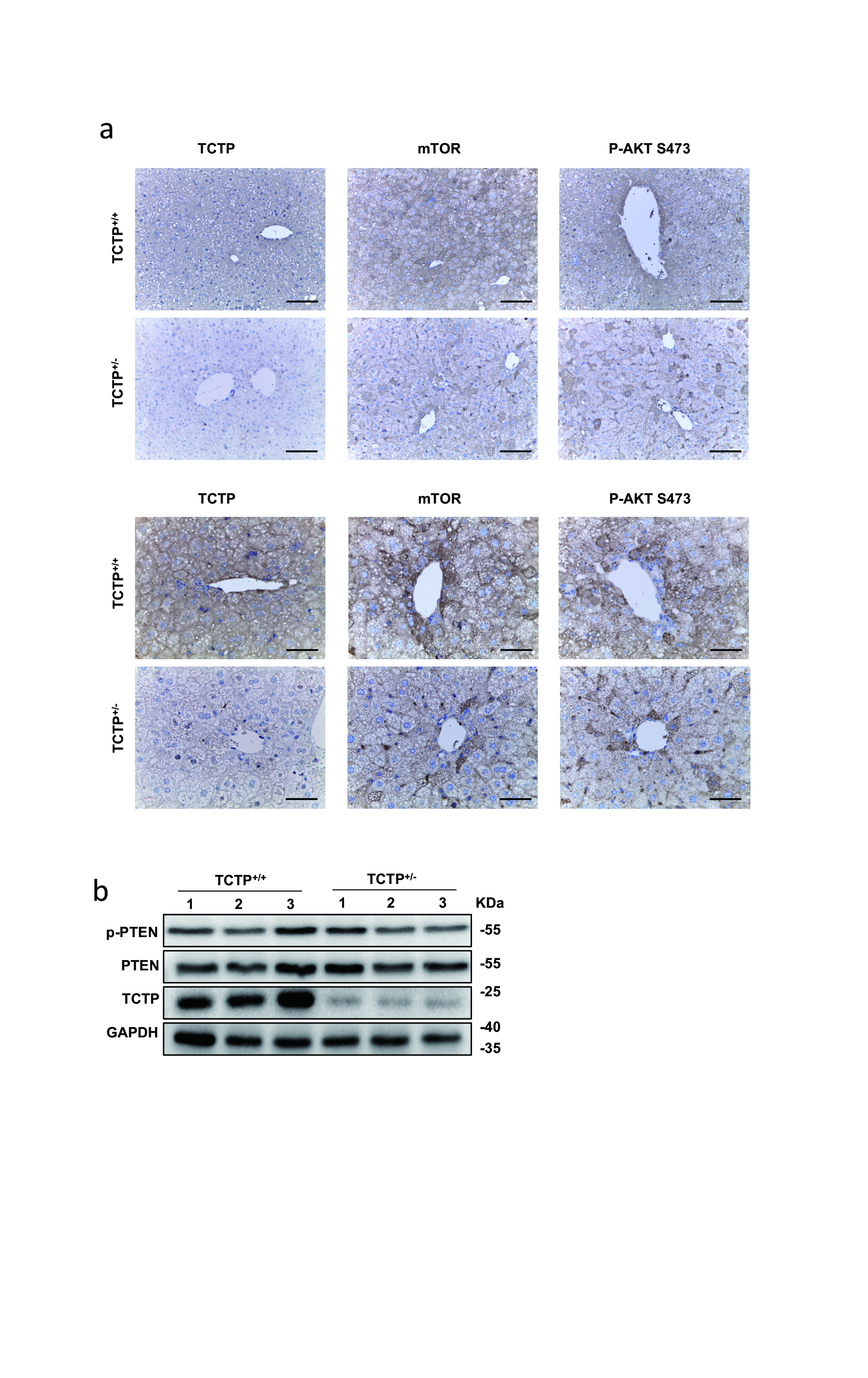

Supplement: Supplementary file 4 — supplementary figure 3 [file 41419_2020_2231_MOESM4_ESM.tif]

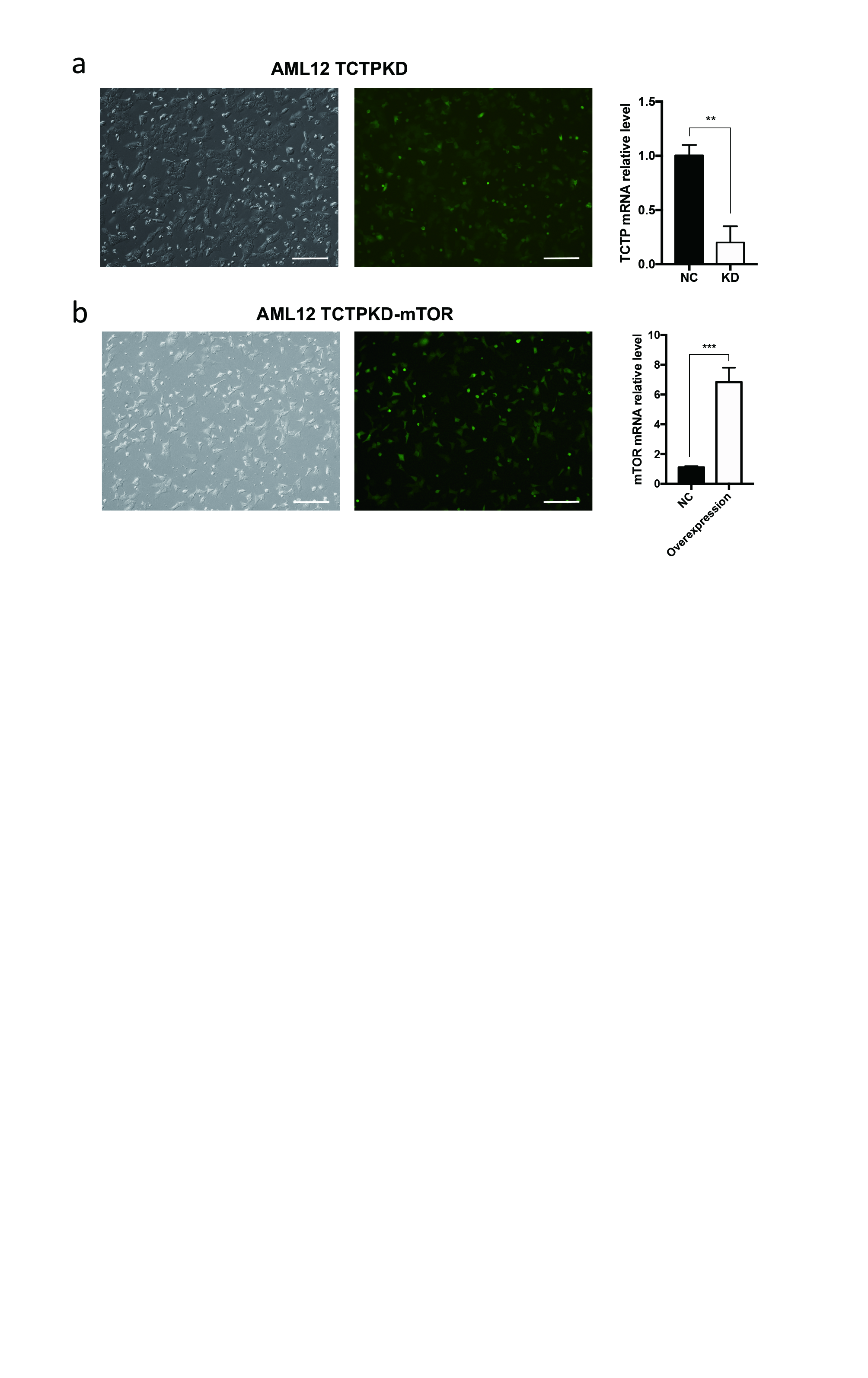

Supplement: Supplementary file 5 — supplementary figure 4 [file 41419_2020_2231_MOESM5_ESM.tif]

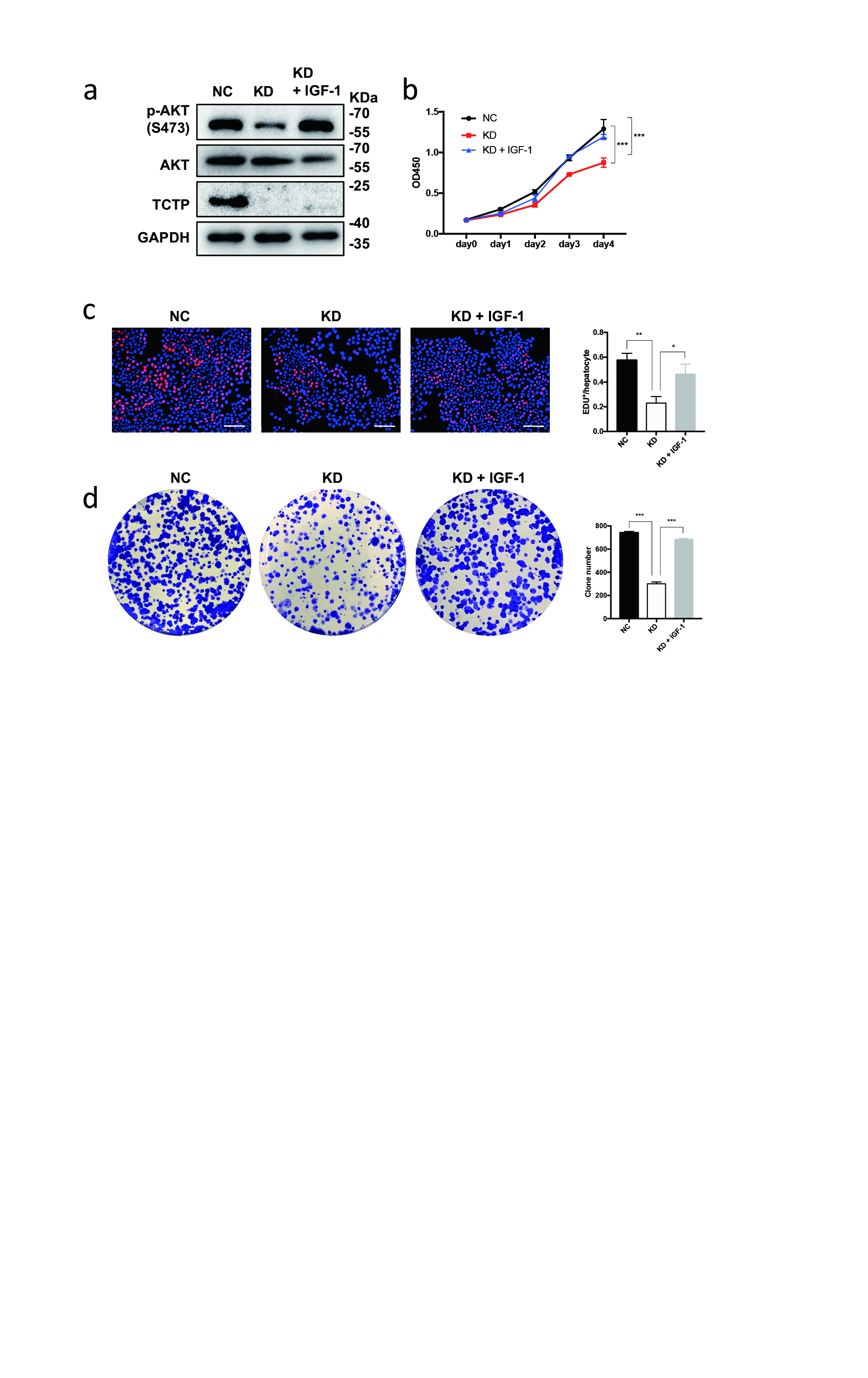

Supplement: Supplementary file 6 — supplementary figure 5 [file 41419_2020_2231_MOESM6_ESM.tif]

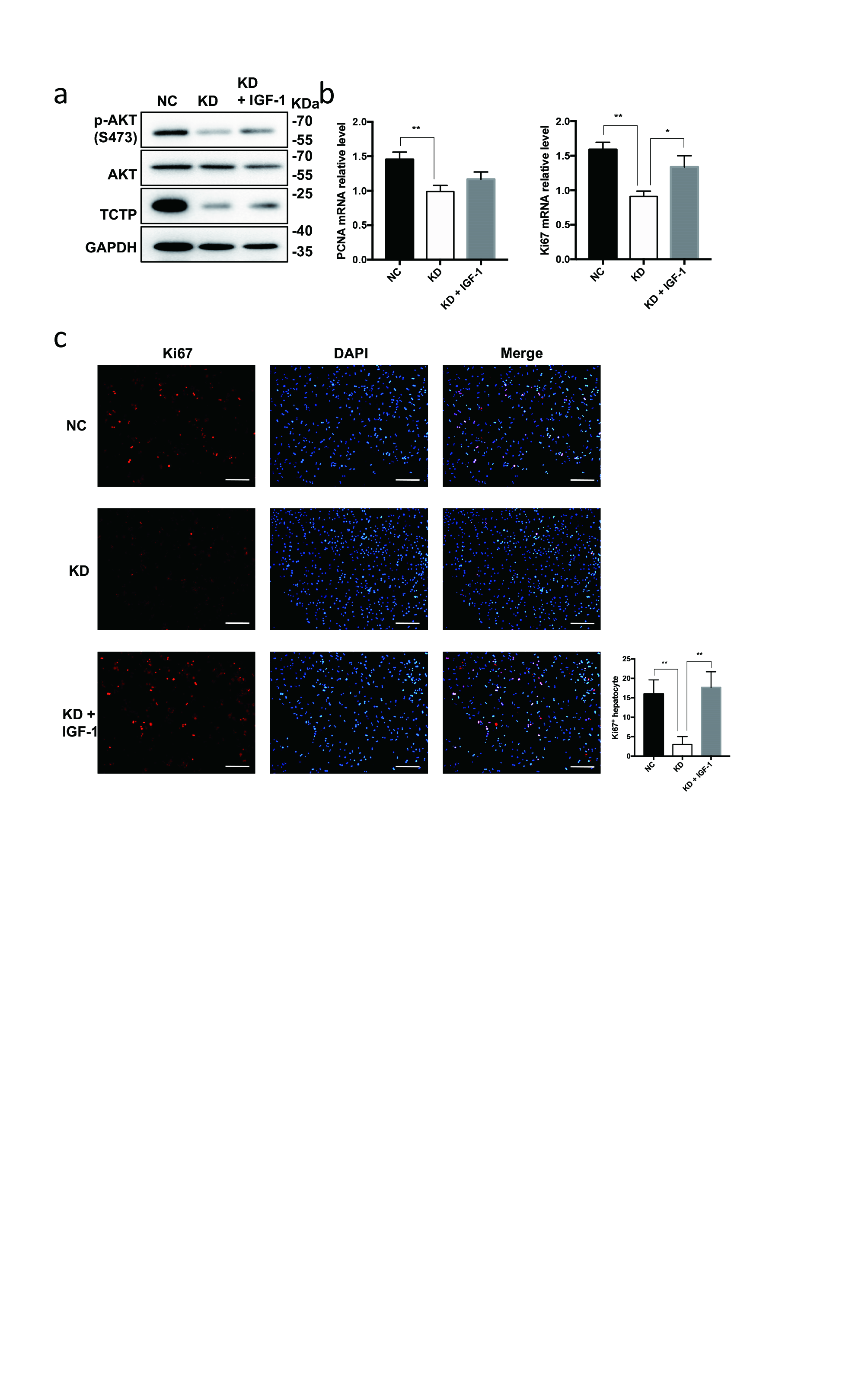

Supplement: Supplementary file 7 — supplementary figure 6 [file 41419_2020_2231_MOESM7_ESM.tif]

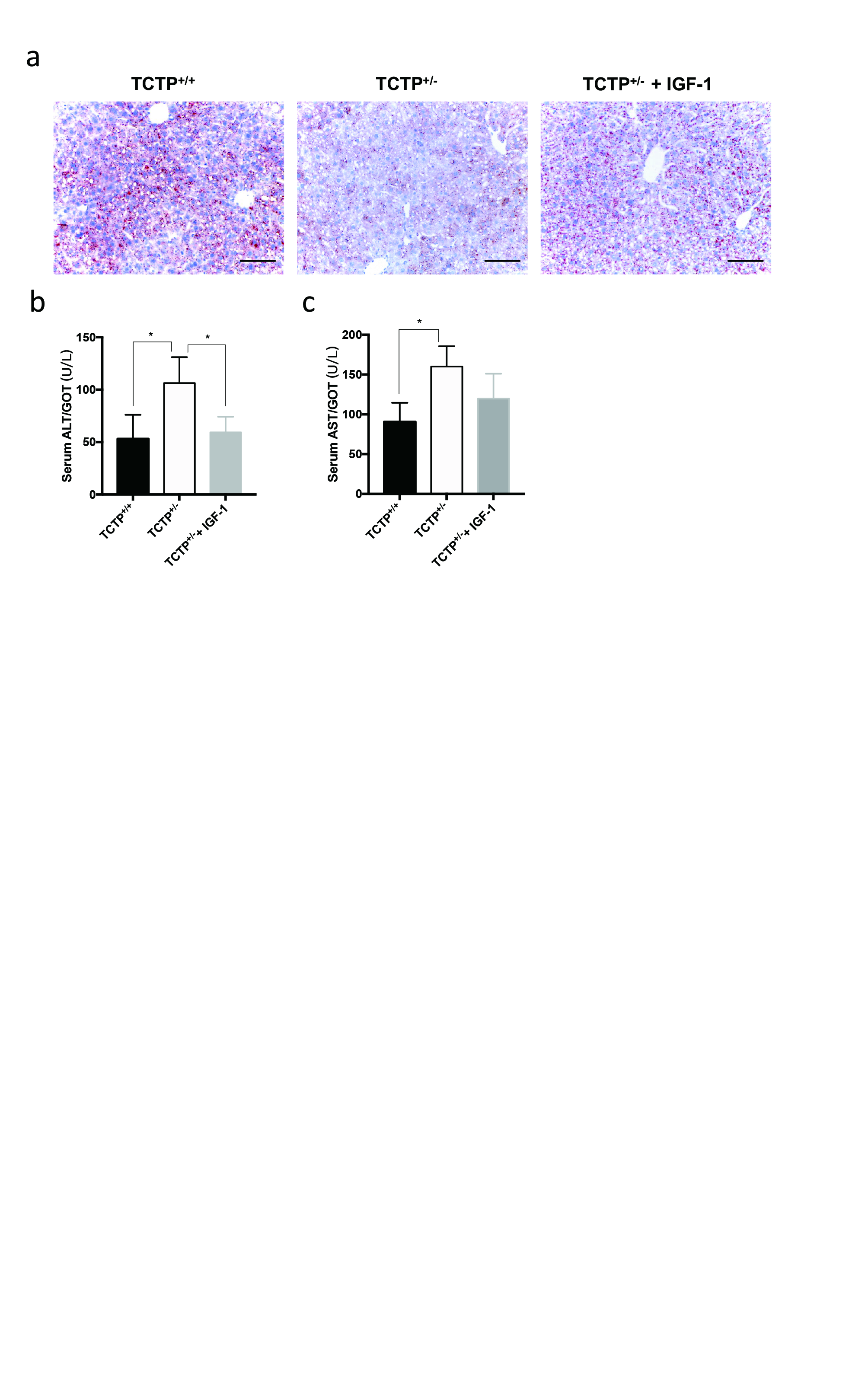

Supplement: Supplementary file 8 — supplementary figure 7 [file 41419_2020_2231_MOESM8_ESM.tif]

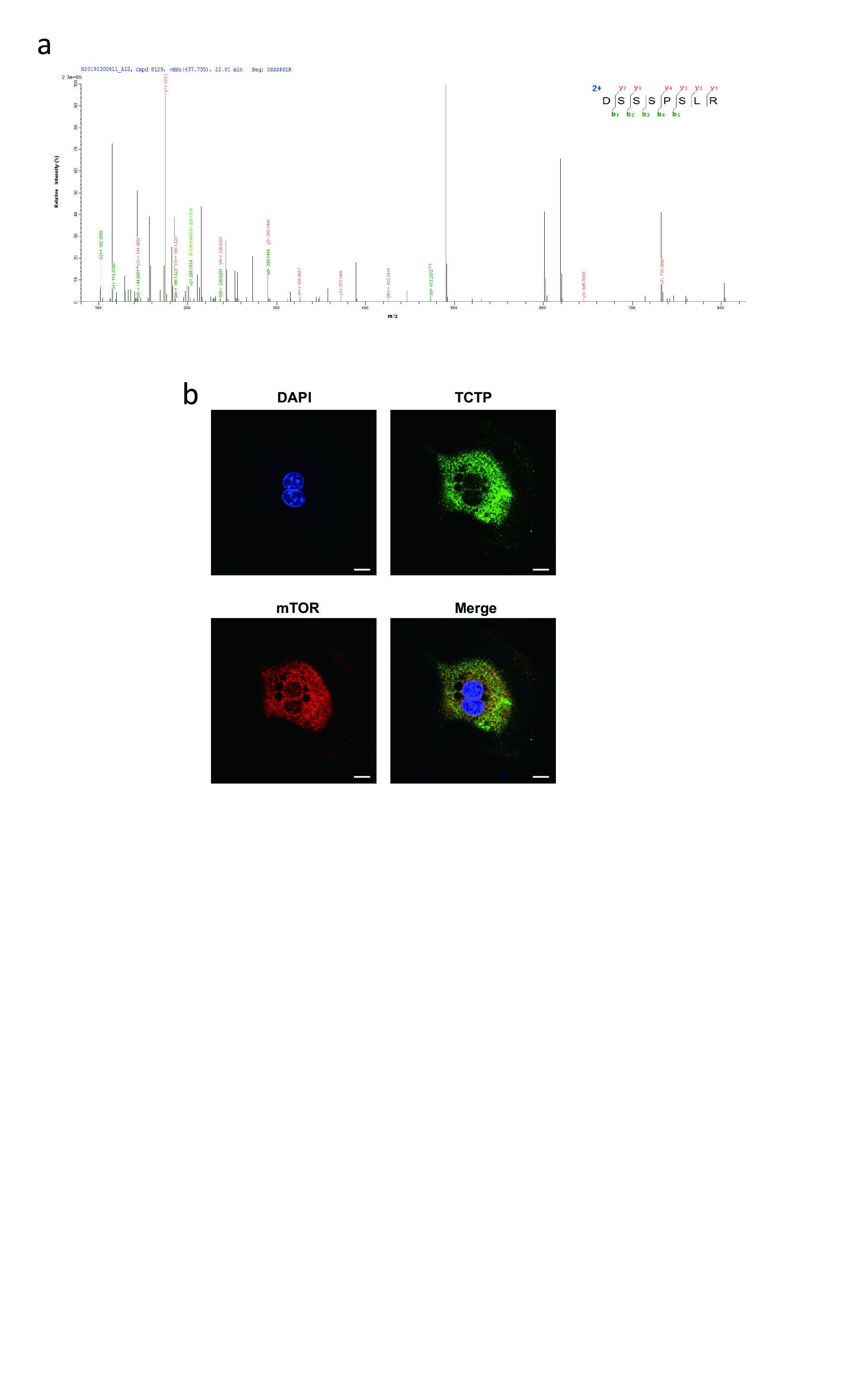

Supplement: Supplementary file 9 — supplementary figure 8 [file 41419_2020_2231_MOESM9_ESM.tif]

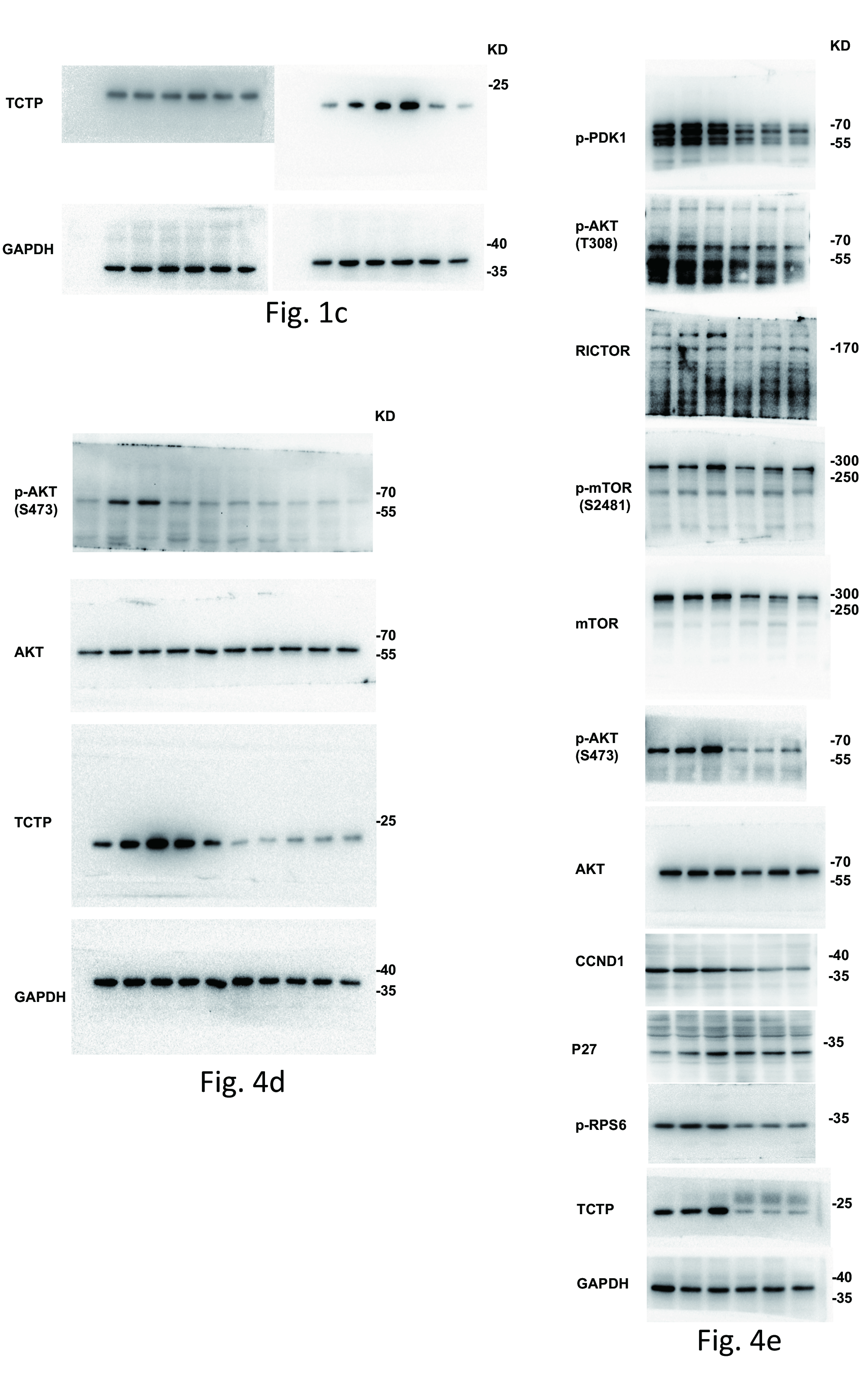

Supplement: Supplementary file 10 — Dataset 1 [file 41419_2020_2231_MOESM10_ESM.tif]

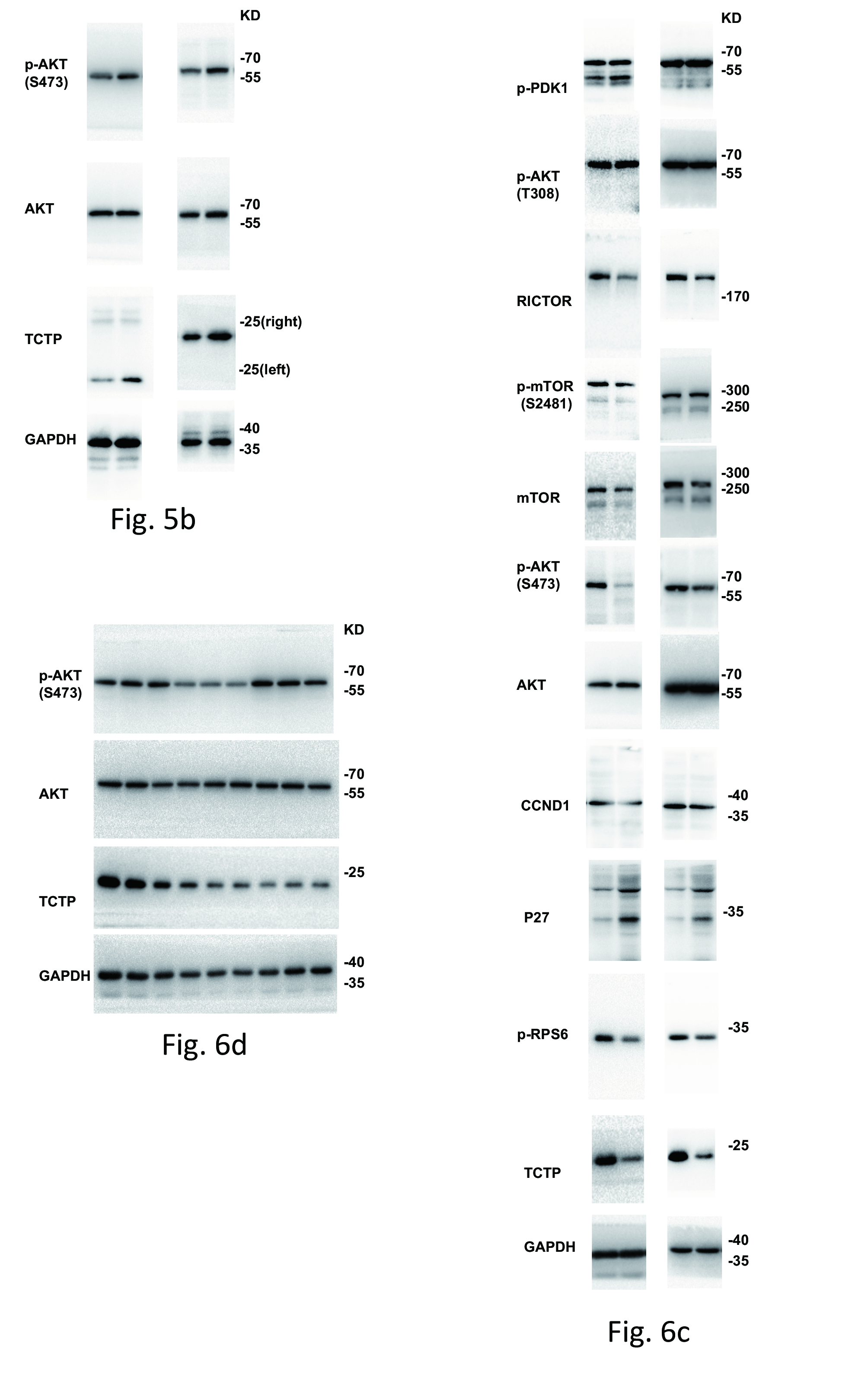

Supplement: Supplementary file 11 — Dataset 2 [file 41419_2020_2231_MOESM11_ESM.tif]

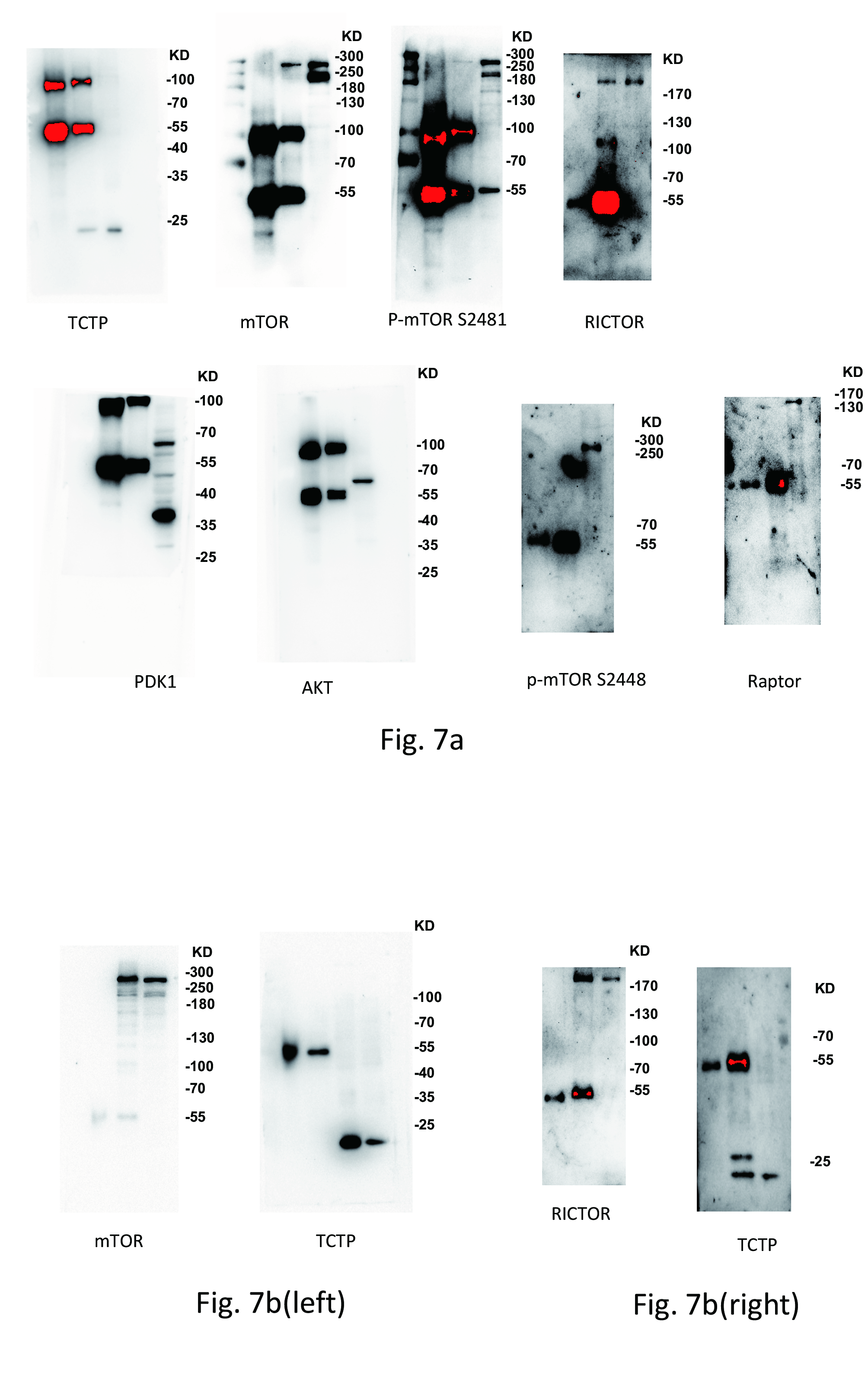

Supplement: Supplementary file 12 — Dataset 3 [file 41419_2020_2231_MOESM12_ESM.tif]

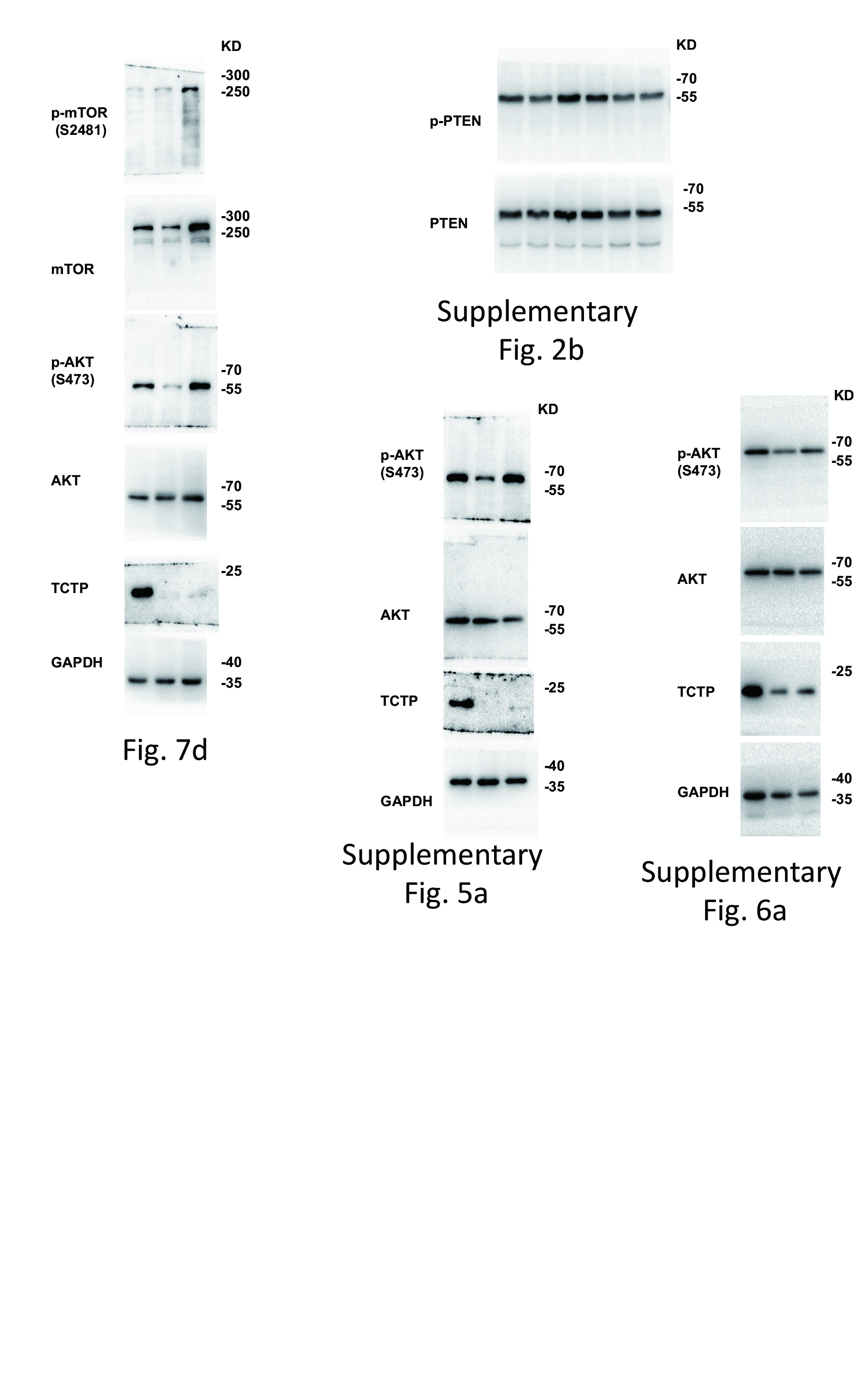

Supplement: Supplementary file 13 — Dataset 4 [file 41419_2020_2231_MOESM13_ESM.tif]
